# Total and H-specific GDF-15 levels increase in caloric deprivation independently of leptin in humans

**DOI:** 10.1038/s41467-024-49366-y

**Published:** 2024-06-18

**Authors:** Pavlina Chrysafi, Laura Valenzuela-Vallejo, Konstantinos Stefanakis, Theodoros Kelesidis, Margery A. Connelly, Christos S. Mantzoros

**Affiliations:** 1 Department of Medicine, Beth-Israel Deaconess Medical Center, Harvard Medical School, Boston, MA 02215 USA; 2 Department of Medicine, David Geffen School of Medicine, UCLA, Los Angeles, CA 02215 USA; 3 Labcorp, Morrisville, NC 27560 USA; 4 Department of Medicine, Boston VA Healthcare System, Boston, MA 90095 USA

**Keywords:** Multihormonal system disorders, Homeostasis

## Abstract

Mitochondrial-secreted growth differentiation factor-15 (GDF-15) promotes weight loss in animals. Its effects in humans remain unclear, due to limited research and potential measurement interference from the H202D-variant. Our post-hoc analysis investigates total (irrespective of genetic variants) and H-specific GDF-15 (detected only in H202D-variant absence) in humans under acute and chronic energy deprivation, examining GDF-15 interaction with leptin (energy homeostasis regulator) and GDF-15 biologic activity modulation by the H202D-variant. Total and H-specific GDF-15 increased with acute starvation, and total GDF-15 increased with chronic energy deprivation, compared with healthy subjects and regardless of leptin repletion. Baseline GDF-15 positively correlated with triglyceride-rich particles and lipoproteins. During acute metabolic stress, GDF-15 associations with metabolites/lipids appeared to differ in subjects with the H202D-variant. Our findings suggest GDF-15 increases with energy deprivation in humans, questioning its proposed weight loss and suggesting its function as a mitokine, reflecting or mediating metabolic stress response.

## Abstract (web summary)

GDF15 is secreted my mitochondria and promotes weight loss in animals, but its effect in humans remain unclear. Here, they authors show that in humans, GDG15 is increase in females with chronic caloric deficit, independent of leptin levels, and that it’s positively correlated with triglyceride-rich particiles.

## Introduction

Growth differentiation factor-15 (GDF-15) is a circulating protein, distant member of the transforming growth factor-β family^1^. GDF-15 acts as a stress-response cytokine/mitokine (mitochondrial stress-induced cytokine) and is upregulated in a broad spectrum of conditions, ranging from endurance exercise to mitochondrial dysfunction, cellular injury, and inflammation^1–3^. In addition, current evidence suggests an involvement of GDF-15 in energy homeostasis. However, the mechanistic pathways underlying the effects of GDF-15 in human metabolism regulation remain unclear^4,5^. Centrally, GDF-15 is known to activate the glial-derived neurotrophic factors receptor-α-like (GFRAL)^6^, which is highly expressed in the hindbrain and has been proposed to mediate appetite suppression and weight loss in mice treated with exogenous GDF-15^7,8^. Recently, animal studies have associated GDF-15 with the induction of appetite and weight loss, cachexia, increased oxidative metabolism, and upregulation of crucial thermogenetic and lipolytic genes^3,7,9–11^.

Whether the physiological effects of GDF-15 observed in animals can be applied to and replicated in humans remains to be elucidated. Despite the development of GDF-15 mimicking compounds for weight loss and the ongoing development of GDF-15 inhibitors against cachexia and conditions of metabolic stress^8,12^. More specifically, the current results on the role of GDF-15 in weight homeostasis in humans are inconsistent, with some studies emphasizing the relationship between GDF-15 and cachexia and others reporting varying roles in obesity^1^. This underscores the lack of conclusive human physiology studies and raises the possibility of unreliable assessment of GDF-15 in humans, possibly due to a systematic bias in GDF-15 measurement^13^, which could be potentially attributed to the existence of genetic variants of the molecule. It has been reported that 15–35% of the population carry a missense variant (Cytosine→Guanine;c.202 C > G) in the GDF-15 coding region, which leads to the substitution of histidine (H) at the 202 aminoacidic residue for aspartic acid (D) during translation of the pro-peptide^13^. This variant may alter the antigenicity of the molecule, which could further interfere with enzyme-linked immunosorbent assay (ELISA) measurements, generate methodological problems, and thus, produce inconclusive study findings^13,14^. Some commonly used commercial immunoassays could underestimate circulating levels of GDF-15 (decreased antibody affinity leading to reduced absorbance readings) in the DD or HD alleles. Therefore, studies need to be cautiously appraised considering genotype differences between groups, and a correction factor may need to be implemented to account for methodological and measurement discrepancies^13–16^.

Possible functional differences within the GDF-15 genetic variants should also be considered. The H202D variant has been proposed to result in biological activity differences, increasing the risk for the development/progression of inflammatory diseases^14,15^. Thus, whether the H202D variant interferes with the accurate measurement of GDF-15, or its biology remains unknown. Most importantly, it remains to be seen what the exact role of GDF-15 in human weight homeostasis is, what the potential discrepancies between animals and humans are, and whether GDF-15 is associated with other weight-regulating hormones, like leptin^17^. We have previously shown that the adipocyte-secreted hormone leptin is downregulated with starvation and mediates the neuroendocrine and metabolic response to acute and chronic energy deprivation^18^, but research on possible interactions between leptin and GDF-15 in energy homeostasis remains limited.

To address these questions, we performed post-hoc analyses to explore the physiological role of GDF-15 during acute complete and chronic mild energy deprivation, with and without leptin administration in lean participants. Chronic energy deprivation was studied in participants with relative energy deficiency in sport syndrome (REDs), previously defined as hypothalamic amenorrhea due to insufficient caloric intake or excessive energy expenditure, altering several physiological and metabolic functions^19–21^. To measure GDF-15, we used two GDF-15 ELISAs. The first assay measures total GDF-15, which can detect GDF-15 irrespective of the presence of the H202D variant^22,23^. The second assay measures H-specific GDF-15, precisely and uniquely detecting the H amino acid at the 202 position in the GDF-15 sequence and does not detect genetic variants generated by the change of the H to D, called H202D; thus, it can detect only GDF-15 without the H202D variant^4,5,13,16^. Secondary aims included exploratory correlations with GDF-15 and lipids, lipoproteins, and metabolites to raise hypotheses on metabolic pathways that may be associated with GDF-15.

## Results

### Baseline clinical and biochemical parameters and GFD-15 levels in lean, healthy humans, and in lean individuals with chronic mild caloric deprivation

Demographic, clinical, and biochemical characteristics of participants are summarized in Table 1. Parametric t-tests evaluated differences based on their sex, study of participation, and presence of the H202D variant. In particular, Studies 1 and 2 were assessed together in Table 1a and independently (i.e., 6 males and 7 female participants from Study-1 in Table 1b and 15 females with REDs in Table 1c).

**Table 1:** a Studies 1 and 2: Clinical and biochemical data of participants at baseline; b Study-1 (3-day intervention): Clinical and biochemical data of participants at baseline; c Study-2 (36-weeks leptin): Clinical and biochemical data of participants at baseline

a									
	Men, *n* = 6	Women, *n* = 22	*p*-value^a^	*p*-value^b^	Total sample, *n* = 28	Without the H202D variant, *n* = 21	With the H202D variant, *n* = 7	*p*-value^c^	*p*-value^d^
Age, years	23.50 ± 1.54	25.36 ± 0.88	0.323	0.661	24.96 ± 0.77	23.95 ± 0.80	27.50 ± 1.55	0.066	0.054
Weight, Kg	76.06 ± 1.72	56.34 ± 1.35	**<0.001**	NA	60.57 ± 1.98	62.72 ± 2.53	55.17 ± 1.94	**0.026**	NA
Fat mass, Kg	12.63 ± 0.80	14.48 ± 0.81	0.278	**<0.001**	14.09 ± 0.71	14.64 ± 0.75	12.69 ± 1.58	0.291	0.776
Lean mass, Kg	63.43 ± 1.27	41.85 ± 1.05	**<0.001**	**<0.001**	46.48 ± 1.95	48.08 ± 2.54	42.48 ± 2.04	0.098	0.776
BMR, Kcal/day	1759.60 ± 58.60	1210.30 ± 30.45	**0.002**	**0.034**	1328.01 ± 53.41	1377.04 ± 69.17	1205.43 ± 55.15	0.064	0.751
Total GDF-15, pg/mL	568.33 ± 29.30	746.22 ± 72.90	**0.032**	0.472	708.12 ± 58.99	605.77 ± 42.66	963.99 ± 146.75	**0.046**	**0.004**
H-specific GDF-15, pg/mL	332.21 ± 74.53	346.08 ± 40.25	0.875	0.617	342.62 ± 34.49	342.62 ± 34.49	NA	NA	NA
HR, bpm	54.20 ± 1.69	55.61 ± 2.42	0.636	0.631	55.31 ± 1.93	55.48 ± 2.34	54.88 ± 3.64	0.890	0.608
SBP, mmHg	122.75 ± 4.47	97.36 ± 2.11	**0.001**	0.133	102.80 ± 2.75	105.11 ± 3.19	97.04 ± 5.13	0.205	0.774
DBP, mmHg	69.94 ± 2.10	58.22 ± 1.61	**0.001**	0.369	60.68 ± 1.61	61.52 ± 1.94	58.58 ± 2.91	0.417	0.910
Leptin, ng/mL	2.64 ± 0.56	6.99 ± 0.99	**0.004**	**0.048**	6.06 ± 0.87	6.25 ± 1.07	5.59 ± 1.54	0.730	0.724
FFA, mmol/L	0.44 ± 0.03	0.32 ± 0.06	0.089	0.425	0.35 ± 0.05	0.37 ± 0.06	0.29 ± 0.07	0.417	0.469
Cortisol, μg/dL	11.89 ± 0.41	18.90 ± 0.76	**<0.001**	**0.040**	17.40 ± 0.82	17.42 ± 0.98	17.33 ± 1.55	0.960	0.260
Aldosterone, pg/mL	76.94 ± 10.71	73.93 ± 6.80	0.818	0.080	74.63 ± 5.69	71.78 ± 5.84	81.03 ± 13.48	0.543	0.938
Free T4, ng/dL	0.87 ± 0.11	1.04 ± 0.58	0.230	0.429	1.00 ± 0.05	1.00 ± 0.06	1.01 ± 0.12	0.960	0.900

b									
	Men, *n* = 6	Women, *n* = 7	*p*-value^e^	*p*-value^f^	Total sample, *n* = 13	Without the H202D variant, *n* = 10	With the H202D variant, *n* = 3	*p*-value^g^	*p*-value^h^
Age, years	23.50 ± 1.54	23.71 ± 1.46	0.922	0.588	23.61 ± 1.01	23.62 ± 1.02	25.00 ± 2.08	0.501	0.734
Weight, Kg	76.06 ± 1.72	56.65 ± 2.33	**<0.001**	NA	65.60 ± 3.33	68.13 ± 3.34	57.21 ± 2.22	**0.036**	NA
Fat mass, Kg	12.63 ± 0.80	17.22 ± 1.44	**0.042**	**<0.001**	15.10 ± 1.16	15.11 ± 1.17	13.38 ± 3.40	0.585	0.918
Lean mass, Kg	63.43 ± 1.27	39.42 ± 1.34	**<0.001**	**<0.001**	50.50 ± 3.66	52.50 ± 3.67	43.83 ± 5.26	0.260	0.917
BMR, Kcal/day	1759.60 ± 58.60	1294.52 ± 51.51	**0.003**	0.609	1509.17 ± 84.58	1570.49 ± 100.43	1304.80 ± 86.95	0.081	0.689
Total GDF-15, pg/mL	568.33 ± 29.30	470.72 ± 51.32	0.132	0.558	515.81 ± 32.74	493.18 ± 37.07	591.25 ± 59.52	0.239	**0.048**
H-specific GDF-15, pg/mL	332.21 ± 74.53	291.54 ± 73.10	0.707	0.986	311.90 ± 49.68	311.90 ± 49.68	NA	NA	NA
HR, bpm	54.20 ± 1.69	65.50 ± 3.88	**0.028**	0.701	60.28 ± 2.69	60.28 ± 2.69	59.00 ± 1.17	0.662	0.131
SBP, mmHg	122.75 ± 4.47	103.00 ± 2.44	**0.005**	0.170	112.11 ± 3.67	112.12 ± 3.68	108.44 ± 4.58	0.489	0.848
DBP, mmHg	69.94 ± 2.10	59.12 ± 2.44	**0.007**	**0.029**	64.00 ± 2.18	64.00 ± 2.18	63.56 ± 5.57	0.931	0.846
Leptin, ng/mL	2.64 ± 0.56	12.62 ± 1.26	**<0.001**	**0.005**	8.01 ± 1.62	8.01 ± 1.62	8.97 ± 3.21	0.759	0.970
FFA, mmol/L	0.44 ± 0.03	0.05 ± 0.01	**<0.001**	**<0.001**	0.23 ± 0.05	0.23 ± 0.06	0.21 ± 0.14	0.835	0.281
Cortisol, μg/dL	11.89 ± 0.41	17.02 ± 1.31	**0.007**	0.157	14.65 ± 1.01	14.65 ± 1.02	13.43 ± 1.52	0.456	0.065
Aldosterone, pg/mL	76.94 ± 10.71	62.96 ± 6.86	0.301	0.304	69.41 ± 6.22	69.42 ± 6.23	69.49 ± 10.50	0.994	0.884
Free T4, ng/dL	0.87 ± 0.11	1.20 ± 0.07	**0.040**	0.522	1.04 ± 0.07	0.94 ± 0.08	1.39 ± 0.05	**<0.001**	**0.021**

c					
	Total sample, *n* = 15	Without the H202D variant, *n* = 11	With the H202D variant, *n* = 4	*p*-value^i^	*p*-value^j^
Age, years	26.13 ± 1.07	24.70 ± 1.06	29.00 ± 1.97	0.101	**0.040**
Weight, Kg	56.20 ± 1.71	57.32 ± 2.14	53.95 ± 2.84	0.370	NA
Fat mass, Kg	13.20 ± 0.82	13.66 ± 0.85	12.28 ± 1.86	0.526	0.814
Lean mass, Kg	42.99 ± 1.33	43.65 ± 1.60	41.66 ± 1.69	0.441	0.814
BMR, Kcal/day	1171.00 ± 34.09	1183.60 ± 42.39	1145.80 ± 62.27	0.630	0.849
Total GDF-15, pg/mL	874.78 ± 86.35	718.35 ± 59.09	1187.63 ± 59.08	**0.040**	**0.005**
H-specific GDF-15, pg/mL	373.33 ± 48.45	373.33 ± 48.45	NA	NA	NA
HR, bpm	51.00 ± 2.27	50.30 ± 2.16	52.40 ± 5.71	0.744	0.771
SBP, mmHg	94.73 ± 2.66	97.00 ± 2.59	90.20 ± 6.01	0.342	0.463
DBP, mmHg	57.80 ± 2.13	58.90 ± 2.60	55.60 ± 3.70	0.438	0.910
Leptin, ng/mL	4.36 ± 0.55	4.77 ± 0.70	3.56 ± 4.77	0.310	0.524
FFA, mmol/L	0.46 ± 0.06	0.49 ± 0.83	0.35 ± 0.08	0.241	0.308
Cortisol, μg/dL	19.78 ± 0.87	19.83 ± 1.10	19.67 ± 1.55	0.935	0.766
Aldosterone, pg/mL	76.83 ± 9.58	74.77 ± 9.37	87.95 ± 21.05	0.590	0.793
Free T4, ng/dL	0.69 ± 0.71	1.05 ± 0.92	0.78 ± 0.92	**0.022**	0.119

Data are presented as mean ± SEM. Two-sided *p*-values are presented, with significance (<0.05) indicated in bold.

*BMR* Basal metabolic rate, *BPM* Beats per minute, *DBP* Diastolic blood pressure, *FFA* Free fatty acids, *GDF-15* Growth differentiation factor-15, *HR* Heart rate, *NA* Not applicable, *SBP* Systolic blood pressure, *T4* Thyroxine

^a^*p*-value from an independent sample *t*-test between males (*n* = 6) and females (*n* = 22).

^b^*p*-value from analysis of covariance (ANCOVA) between males (*n* = 6) and females (*n* = 22), adjusted by the characteristic: Body Weight.

^c^*p*-value from an independent sample *t*-test between subjects with (*n* = 7) and without the H202D variant (*n* = 21).

^d^*p*-value from ANCOVA between subjects with (*n* = 7) and without the H202D variant (*n* = 21), adjusted by the characteristic: Gender + Body Weight

^e^*p*-value from an independent sample *t*-test between males (*n* = 6) and females (*n* = 7).

^f^*p*-value from an analysis of covariance (ANCOVA) between males (*n* = 6) and females (*n* = 7), adjusted by the characteristic: Body Weight.

^g^*p*-value from an independent sample *t*-test between subjects with (*n* = 3) and without the GDF-15 variant (*n* = 10).

^h^*p*-value from an ANCOVA between subjects with (*n* = 3) and without the GDF-15 variant (*n* = 10), adjusted by the characteristic: Gender + Body Weight.

^i^*p*-value from an independent sample *t*-test between subjects with (*n* = 4) without the variant (*n* = 11).

^j^*p*-value from an ANCOVA between subjects with (*n* = 4) and without the GDF-15 variant (*n* = 11) present, adjusted by the characteristic: Body Weight.

**Fig. 1:**
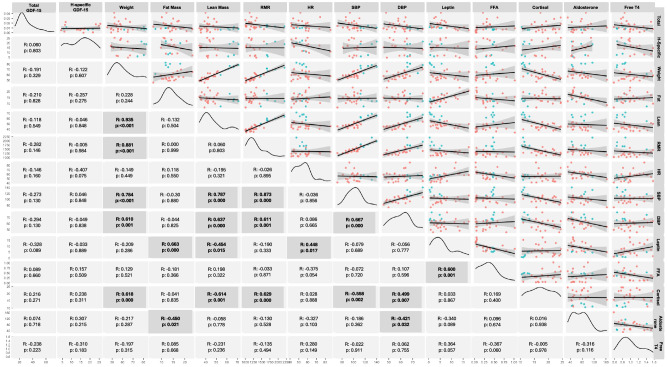
Correlation matrix in lean individuals at baseline (Study-1 and Study-2; *n* = 28). For this analysis, baseline values from participants in Study-1 and Study-2 were used. Correlation matrix was generated in R studio using the Ggally package, and correlation R coefficient and two-sided *p*-value were calculated with Pearson’s Correlations. R code is available at Supplementary Appendix 1. Red dots correspond to female (*n* = 22), and blue dots to male participants (*n* = 6). GDF-15 Growth differentiation factor 15, RMR Resting metabolic rate, HR Heart rate, SBP Systolic blood pressure, DBP Diastolic blood pressure, FFA free fatty acids, T4 Thyroxine.

**Fig. 2:**
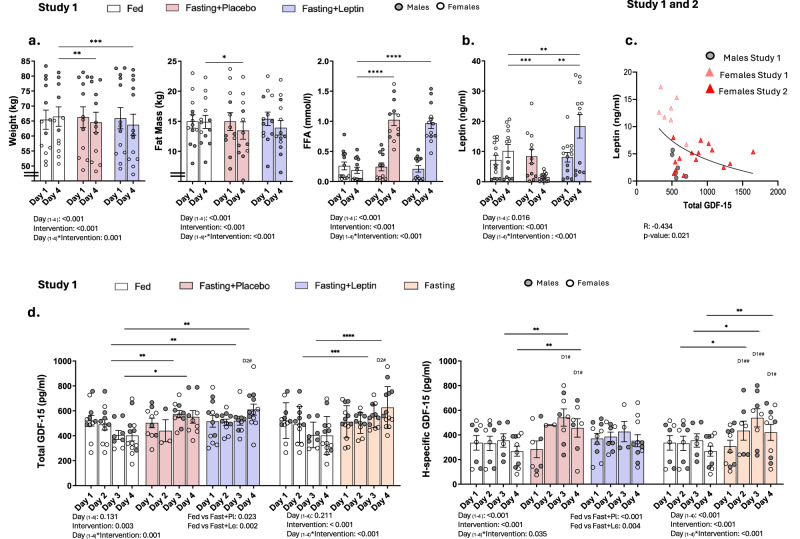
Total and H-specific GDF-15 in response to acute complete caloric deprivation with or without leptin replacement in lean participants. **a** Study-1: A cross-over study in lean participants (*n* = 13) during 3 interventions, i.e., 3-day fed, fasting+placebo, fasting+leptin. Weight, fat mass, and free fatty acids (FFA) change over time. The error bars represent the standard error of the mean. Two-sided *p*-values were calculated by linear mixed models (MM) analyses adjusted for baseline. Factors for MM were Day, i.e., 1,2,3,4, and Intervention, i.e., fed, fasting+placebo, fasting+leptin. By Day _(1-4)_*Intervention <0.05, a least significant difference (LSD) post hoc test was performed (only significant *p*-values are presented). One, two, or three asterisks indicate two-sided *p* < 0.05, <0.01, <0.001. **b** Leptin changes in Study-1 (*n* = 13). The error bars represent the standard error of the mean. Two-sided *p*-values were calculated with MM and post hoc analyses as described above. **c** Baseline total GDF-15 in correlation with baseline leptin levels from Studies 1 and 2. For Study-1, baseline values for each participant were derived from the average of the baselines of the three interventions i.e., fed, fasting+placebo, fasting+leptin. For this analysis only, baseline values from Study-2 (36-weeks leptin) were also included to increase the sample size (total *n* = 28). A curve fit estimation was conducted on SPSS along with the calculation of the ANOVA R coefficient and two-sided *p*-value. **d** Total and H-specific GDF-15 in Study-1 (*n* = 13) during the 3 different interventions, i.e., fed, fasting+placebo, fasting+leptin. The error bars represent the standard error of the mean. MM analysis and post hoc test were analyzed as above. In addition, paired *t*-tests were conducted within each intervention; only significant *p*-values are demonstrated with #Dx where # indicates two-sided *p* < 0.05, and Dx indicates the day within the intervention (i.e., day 1, 2, 3, or 4) from which there was a difference. This analysis was replicated by combining the 2 fasting interventions in one group i,e., averages of each participant from fasting + placebo and fasting + leptin at the corresponding timepoints) and comparing it with the fed state. GDF-15 Growth differentiation factor 15, FFA Free fatty acids.

H-specific GDF-15 was not detected in seven participants. This was attributed to the presence of the H202D variant, given that H-specific is only detected in the absence of the H202D variant. Those seven participants were found to have significantly higher total GDF-15 levels compared to participants without the H202D variant, even after adjusting for body weight and biological sex effects through analyses of covariance (ANCOVA) (Table 1a). This association was mainly observed in Study 2 participants with REDs. Specifically, females with chronic mild energy deficiency in the context of REDs (Study-2) had significantly higher total GDF-15 levels compared to healthy lean women and men of Study-1 (two-sided *p*-value: 0.002). However, this observation was not seen in healthy lean participants from Study 1, who demonstrated similar levels of total GDF-15 despite the presence of the H202D variant (Table 1b). We also performed non-parametric u-tests without major differences from the parametric tests (Supplementary Table 1). These analyses were again evaluated based on participants’ sex, the study of participation, and the presence of the H202D variant. We further performed an exploratory correlation analysis of total and H-specific GDF-15 with Table 1 parameters (Fig. 1). We ascertained a negative association trend between leptin and total GFD-15 through our initial correlation analysis (Fig. 1). This was confirmed by a mild, albeit significant negative association of leptin and total GDF-15 through a logarithmic curve (Fig. 2c).

### Total and H-specific GDF-15 in response to acute complete fasting with and without leptin replacement for 3 days in lean, healthy male and female individuals (Study-1)

In the first interventional study (Study-1), each participant was evaluated under three different conditions: (a) 3-day fed state, (b) 3-day complete fasting treated with placebo (fasting+placebo), and (c) 3-day complete fasting treated with leptin (fasting+leptin). As expected and previously demonstrated^24^, weight and leptin significantly decreased in fasting+placebo, and free fatty acids (FFA) increased in response to fasting independent of leptin (Fig. 2a, b). Compared to the fed state, acute complete starvation increased total (*p*-day*intervention = 0.001 (fed day-3 vs fasting day-3 *p* = 0.001, fed day-4 vs. fasting day-4 *p* = 0.027) and H-specific GDF-15 (*p*-day*intervention = 0.001 (fed day-3 vs. fasting day-3 *p* = 0.008, fed day-4 vs. fasting day-4 *p* = 0.002)). However, leptin administration during fasting (fasting+leptin group) did not affect total and H-specific GDF-15 levels when compared with placebo (fasting+placebo group). This suggests that leptin replacement during fasting does not restore GDF-15 levels to baseline, hence proposing a leptin-independent GDF-15 response to starvation (Fig. 2d).

Given the lack of differences between the placebo and leptin groups during fasting, we proceeded with combining both fasting intervention arms into one single group, to be compared with the isocaloric fed state. In agreement with the above findings (when evaluating fasting+placebo or fasting+leptin separately), by combining both fasting interventions, total and H-specific GDF-15 significantly increased with fasting, an effect that became more pronounced after 48 h of starvation. Total GDF-15 levels progressively increased until the final day of fasting, whereas H-specific GDF-15 levels started to plateau on the final day but remained higher than baseline (Fig. 2d).

### Total and H-specific GDF-15 in response to long-term leptin administration vs. placebo in lean female individuals with chronic relative energy deficiency (Study-2)

**Fig. 3:**
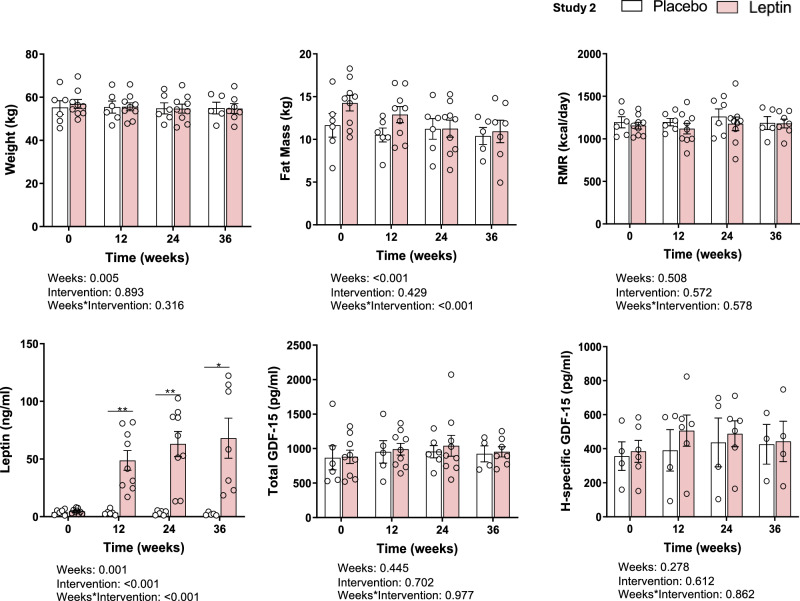
Total and H-specific GDF-15 in response to chronic mild caloric deprivation with leptin or placebo administration in lean females (Study-2; *n* = 15). Weight, fat mass, resting metabolic rate (RMR), leptin, total and H-specific GDF-15. The error bars represent the standard error of the mean. Two-sided *p*-values were calculated using mixed models (MM) analyses adjusted for baseline. Factors for MM were Week, i.e., 0, 12, 24, 32, and Intervention, i.e., leptin and placebo. By Day*Intervention with a *p* < 0.05, a least significant difference (LSD) post hoc test was performed (only significant *p*-values are presented). One, two, or three asterisks indicate two-sided *p* < 0.05, <0.01, <0.001. GDF-15 Growth differentiation factor 15.

To explore the potential long-term effects of leptin on GDF-15 levels in female individuals with chronic mild energy deficiency due to REDs with hypothalamic amenorrhea and hypoleptinemia. All females had low but stable body weight for at least 6 months before the study and were randomly allocated to placebo or leptin administration for 36 weeks. Throughout the treatment, leptin doses were adjusted accordingly to prevent weight loss for each participant, as previously described^25^. For instance, leptin administration replaced leptin levels back to supra-physiological levels^19^. No time- or group-related differences in total or H-specific GDF-15 were ever observed over the course of the study in either treatment group, reaffirming, in the long-term, the leptin-independent action of GDF-15 that was observed acutely in Study-1 (Fig. 3).

### Association of total and H-specific GDF-15 with markers of the metabolic and lipid panel in lean individuals at baseline

As secondary aims, to further explore any potential implication of GDF-15 in metabolic pathways, we performed exploratory correlations of total and H-specific GDF-15 at baseline with lipids and metabolites from Study-1 and 2. To increase the strength of these exploratory correlations, we used two different techniques. First, 230 lipids and metabolites were measured with nuclear magnetic resonance (NMR)-based Nightingale technology (Supplementary Table 2a), and additionally, 60 lipids and metabolites were measured using the latest LipoProfile panel from Labcorp Inc (Supplementary Table 2b), which provides more granularity. Correlations were adjusted by the presence of the GDF-15 H202D variant, and the ones that remained significant after the adjustment are presented with the symbol †.

**Fig. 4:**
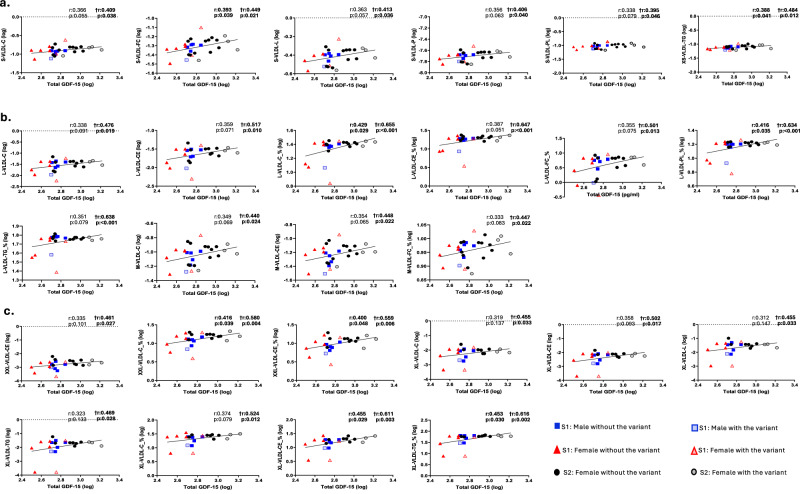
Correlations of total GDF-15 with Nightingale very low-density lipoproteins (VLDL) at baseline (Study-1 and 2; *n* = 28). After logarithmic transformation, Pearson’s correlations and partial correlations adjusted by the presence of the H202D variant were performed between total GDF-15 with Nightingale lipid and metabolite particles at baseline. For the analysis, baseline values from participants in both Study-1 and Study-2 were used and combined in one analysis. For Study-1, since each participant underwent three separate interventions, we used the average of the three intervention baselines as a baseline value. R coefficient and p-value indicate Pearson’s correlations, while the ones marked with † indicate the partial correlations adjusted by the presence of the H202D variant. Significant two-sided *p*-values (<0.05) are in bold. **a** Small and very small VLDL-related particles, **b** Large and medium VLDL-related particles, **c** Extremely and very large VLDL-related particles. GDF-15 Growth differentiation factor 15, L-VLDL-C Total cholesterol in large VLDL, L-VLDL-CE Cholesterol esters in large VLDL, L-VLDL-C_% Total cholesterol to total lipids ratio in large VLDL, L-VLDL-CE_% Cholesterol esters to total lipids ratio in large VLDL, L-VLDL-TG_% Triglycerides to total lipids ratio in large VLDL, L-VLDL-FC_% Free cholesterol to total lipids ratio in large VLDL, M-VLDL-FC_% Free cholesterol to total lipids ratio in medium VLDL, M-VLDL-C Cholesterol in medium VLDL, M-VLDL-CE Cholesterol esters in medium VLDL, S Study, S-VLDL-FC Free cholesterol in small VLDL, S-VLDL-L Total lipids in small VLDL, S-VLDL-P Concentration of small VLDL particles, S-VLDL-PL Phospholipids in small VLDL, XL-VLDL-C Total cholesterol in very large VLDL, XL-VLDL-CE Cholesterol esters in very large VLDL, XL-VLDL-L Total lipids in very large VLDL, XL-VLDL-TG Triglycerides in very large VLDL, XL-VLDL-C_% Total cholesterol to total lipids ratio in very large VLDL, XL-VLDL-CE_% Cholesterol esters to total lipids ratio in very large VLDL, XL-VLDL-TG_% Triglycerides to total lipids ratio in very large VLDL, XS-VLDL-TG Triglycerides in very small VLDL; L-VLDL-PL_% Phospholipids to total lipids ratio in large VLDL, XXL-VLDL-C_% Total cholesterol to total lipids ratio in chylomicrons and extremely large VLDL, XXL-VLDL-CE Cholesterol esters in chylomicrons and extremely large VLDL, XXL-VLDL-CE_% Cholesterol esters to total lipids ratio in chylomicrons and extremely large VLDL.

**Fig. 5:**
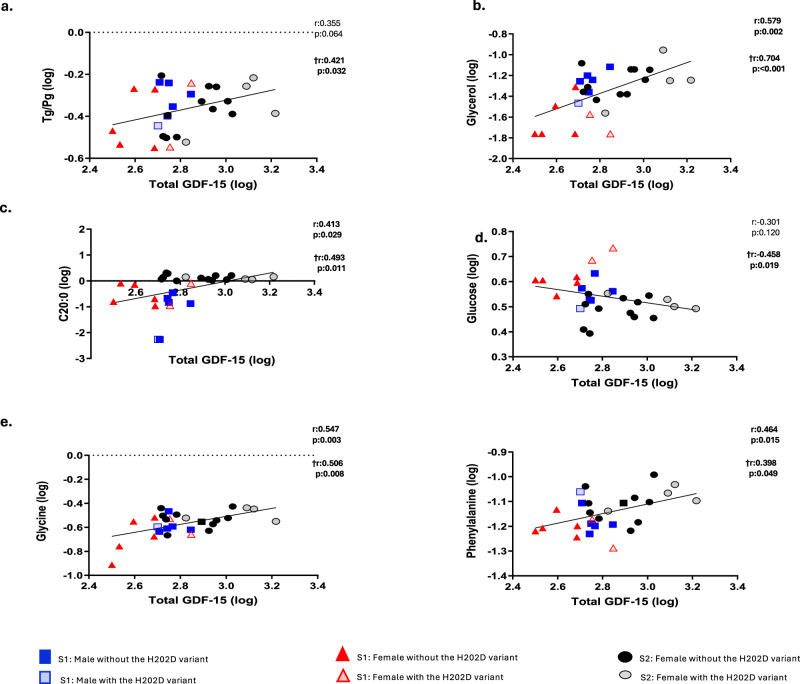
Correlations of total GDF-15 with Nightingale metabolites and lipids at baseline (Study-1 and 2; *n* = 28). After logarithmic transformation, Pearson’s correlations and partial correlations adjusted by the presence of the H202D variant were performed between total GDF-15 with Nightingale lipid and metabolite particles at baseline. For the analysis, baseline values from participants in both Study-1 and Study-2 were used and combined in one analysis. For Study-1, since each participant underwent three separate interventions, we used as baseline value the average of the three intervention baselines. R coefficient and two-sided p-values indicate Pearson’s correlations, while the ones marked with † indicate the partial correlations adjusted by the presence of the variant. Significant two-sided *p*-values (<0.05) are in bold. **a** Tg/Pg, **b** Glycerol, **c** Fatty acids, **d** Glucose, **e** Amino acids. C20:0 arachidic acid, GDF-15 Growth differentiation factor 15, S Study, Tg/Pg Triglycerides to phosphoglycerides ratio.

Our analysis with Nightingale technology demonstrated a positive correlation of total GDF-15 with very low-density lipoproteins (VLDL) -triglyceride-rich particles- and their ratios with other lipid particles (Fig. 4). Particularly, small and very small VLDL particles (Fig. 4a), large and medium VLDL particles (Fig. 4b) and extremely large and very large VDLD particles (Fig. 4c), and their ratios to total cholesterol, cholesterol esters, free cholesterol, phospholipids to total lipids ratio and chylomicrons. Total GDF-15 was also positively correlated with the triglycerides to phosphoglycerides ratio and glycerol (Fig. 5a, b), a component of triglyceride synthesis. Besides, it also displayed a positive correlation with the amino acids glycine and phenylalanine, and the fatty acid C20:0 (arachidic acid) (Fig. 5c, e). Its correlation with glucose was negative (Fig. 5d). These associations were significant after the adjustment for the presence of the GDF-15 H202D variant.

**Fig. 6:**
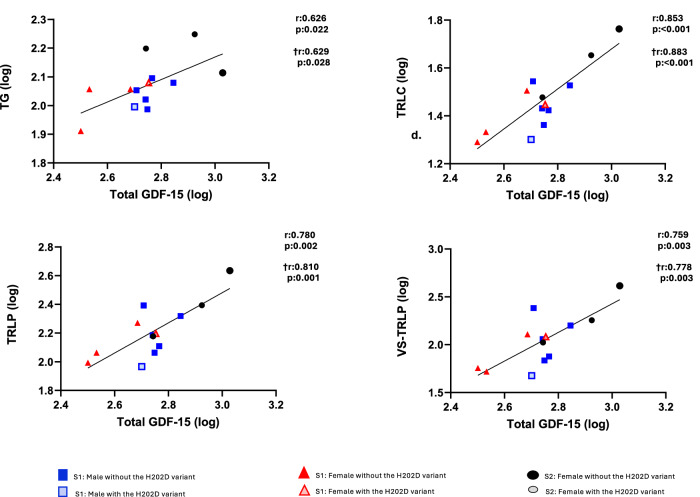
Correlations of total GDF-15 with Labcorp triglyceride-rich particles at baseline (Study-1 and 2; *n* = 28). After logarithmic transformation, Pearson’s correlations and partial correlations adjusted by the presence of the H202D variant were performed between total GDF-15 with Labcorp lipid and metabolite particles at baseline. For Study-1, since each participant underwent three separate interventions, we used as baseline value the average of the three intervention baselines. Correlations with the † symbol are partial correlations adjusted by the presence of the H202D variant. Significant two-sided *p*-values (<0.05) are in bold. GDF-15 Growth differentiation factor 15, S Study, TG Total triglyceride-rich lipoproteins (TLP), TRLC TRL Cholesterol, TRLP triglyceride-rich lipoproteins particles -total chylomicrons and VLDL-, VS-TRLP very small TRL.

Upon replicating our analysis using Labcorp technology, the findings were concordant with those previously mentioned obtained using Nightingale, as shown in Fig. 6. Analysis of Labcorp measurements confirmed the associations above between triglyceride-rich particles and total GDF-15, particularly between total GDF-15 and triglyceride-rich lipoprotein particles in triglycerides and cholesterol (Fig. 6). These associations remained significant after the adjustment for the presence of the GDF-15 H202D variant.

To account for multiple tests, we corrected both analyses by applying the Benjamini–Hochberg false discovery rate (FDR) correction (Supplementary Table 3). Furthermore, for the analyses included in Figs. 4 to 6, we also performed study group-specific and cumulative sensitivity analyses, with and without adjustment for group status (Study-1 and Study-2). Effect modification was assessed using a covariate dummy variable indicating the study group. We found no appreciable differences in around 90% of the variables for the first analyses with Nightingale and Labcorp variables. The variables that showed significant differences between Study-1 and Study-2 are shown in Supplementary Figs. 3 to 5.

Association of changes in total and H-specific GDF-15 with markers of the metabolic and lipid panel in lean individuals measured as delta differences after 3 days acute fasting in Study-1.

We performed Pearson’s exploratory correlations of the changes in total and H-specific GDF-15 with the corresponding changes in metabolites, lipids, and lipoproteins after 3 days of complete fasting under placebo or leptin administration in Study-1 following logarithmic transformation. Our analysis demonstrated that fasting delta changes in total GDF-15 are mainly positively correlated with upregulation in high-density lipoproteins (HDL) particles, while H-specific changes with free and esterified cholesterol, total phosphoglycerides, and cholines. Additionally, H-specific GDF-15 changes during fasting were negatively correlated with respective changes in glucose, acetate, and the ratio of triglyceride to total lipids in very small VLDL (Supplementary Fig. 1). During complete fasting and leptin administration, a positive correlation between arachidonic acid (C20:4n6), α-linolenic acid (C18:3n3), and glycoprotein acetyls, mainly a1-acid glycoprotein with total GDF-15 was seen. Regarding H-specific GDF-15 changes, a positive correlation was seen with changes in triglycerides, glycerol, triglycerides in very small VLDL, and albumin (Supplementary Fig. 2).

### Metabolite and lipid profile comparison between participants with and without the H202D variant

**Fig. 7:**
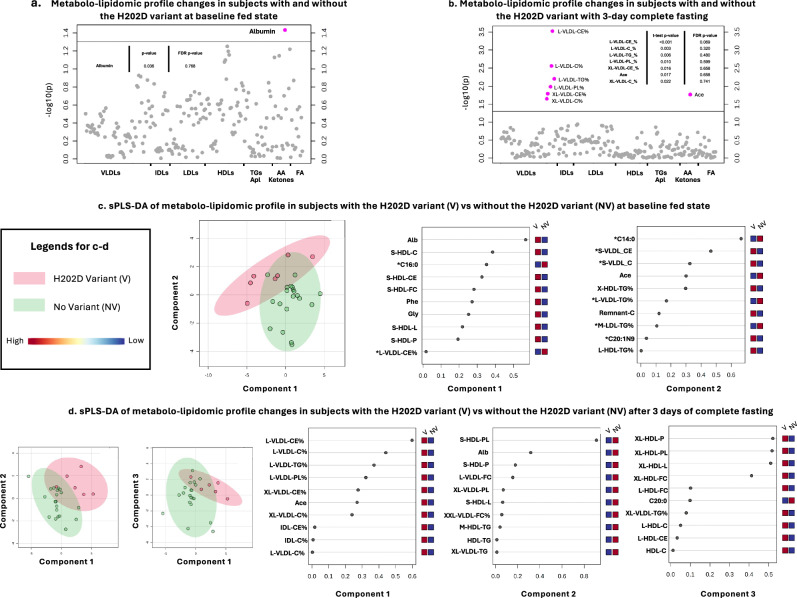
Metabolite-lipid-lipoprotein profile comparison between participants with and without the variant (Study-1 and 2; *n* = 28). Evaluation of metabolites, lipids, and lipoproteins between subjects with and without the variant at baseline fed conditions (**a**; *n* = 28) and after 3 days of acute complete starvation (**b**; *n* = 13). Two-sided *p*-values were calculated with *t*-test and false discovery rate (FDR) correction. sparse Partial Least Squares Discriminant Analysis (sPLS-DA) for presence of the variant (pink) vs. absence of the variant (green) and the parameters that compose each component, their level of contribution (loading) and comparison of their levels between the presence of the H202D variant (V) and absence of the variant (NV) (red color corresponds to higher levels and blue to lower) at baseline fed conditions (**c**; *n* = 28) and after 3 days of acute complete starvation (**d**; *n* = 13). For the analysis in **a** and **c**, we combined Study-1 (average of the three baselines of each participant) and Study-2 at baseline. For the analysis of **b** and **d**, only participants of Study-1 were included. Results from all three interventions (i.e., 3-day fed, fasting+placebo, fasting+leptin) were included as deltas between day 1 and day 4 of each intervention. VLDL Very-low-density, IDL Intermediate-density, LDL Low-density, HDL High-density lipoproteins, TG Triglycerides, Apl Apolipoproteins, AA Amino-acids, FA Fatty-acids, Ace Acetate, Phe Phenylalanine, Gly Glycine, C16:0 Palmitic-acid, C14:0 Miristic-acid, C20:1n9: 11-Eicosenoic-acid, C20:0 Arachidic-acid. The lipid nomenclature categorizes parameters based on both lipoprotein size and composition. The prefixes XL, L, M, and S indicate the size, denoting very large, large, medium, and small lipoproteins, respectively. Meanwhile, the postfixes denote composition: C for total cholesterol, CE for cholesterol esters, PL for phospholipids, and FC for free cholesterol. The percentage value signifies the ratio of the specific component to the total lipoprotein content.

To investigate further the above findings and to explore any potential underlying differences in the bioactivity of total and H-specific GDF-15, we compared circulating metabolomic and lipidomic profiles between participants with and without the mutated variant. At the isocaloric fed state, albumin was the only molecule that demonstrated a significant difference between individuals with and without the H202D variant. Still, this effect was merely suggestive as it was not maintained after FDR correction (Fig. 7a). Several VLDL particles demonstrated a significant difference between individuals with and without the H202D variant following 3 days of complete fasting (Fig. 7b). Although individuals with the H202D variant demonstrated substantially larger changes in the consistency of very large and large VLDL and acetate compared to individuals without it (Fig. 7b); this effect was not maintained after FDR correction (Fig. 7b).

Sparse Partial Least Squares Discriminant Analyses (sPLS-DA) were performed to attempt to distinguish the responses of individuals with and without the H202D variant during fed and fasting states. First, during the fed state, there was distinct clustering and differentiation of participants with and without the H202D variant (Fig. 7c). The main molecular drivers of these differentiations, as seen through the variable importance (VIP) scores, were HDLs in Component-1, and VLDLs in Component-2 (Fig. 7c). Both components included markers that are positively associated with cardiovascular and metabolic diseases (asterisks in Fig. 7c), some of them being higher in the participants with the H202D variant and some of them higher in the group of participants without the H202D variant. Secondly, towards the end of the fasting state, sPLS-DA was again able to discriminate between the two groups, with Component-1 consisting of VLDLs, and Component-3 consisting of HDLs (Fig. 7d). Thus, our analysis reveals clusters of HDL and VLDL-based lipids that may be able to discriminate between participants with the H202D variant.

## Discussion

The primary aim of our study was to explore the changes in the circulating levels and, by extension, establish a potential role of total or H-specific GDF-15 in energy homeostasis, as well as examine the potential role of leptin in mediating these effects. Thus, we performed post-hoc analyses utilizing samples from our previously published mechanistic studies in humans^18,19,26^ to investigate total and H-specific GDF-15 in response to acute complete caloric deprivation with and without leptin replacement, and we compared GDF-15 levels between healthy lean participants and participants with chronic mild caloric deficit, i.e., REDs. We utilized GDF-15 ELISAs that have been previously validated by independent groups in previous studies^4,5,16,23^. Our results demonstrate that total and H-specific GDF-15 increase in response to acute complete starvation and are higher in people with REDs, both independently of leptin. Moreover, as secondary aims, we report correlations of total GDF-15 at baseline, mainly with triglyceride-rich lipids and lipoproteins, as well as a different association with the lipid/metabolite response to acute caloric deprivation in participants with the H202D GDF-15 variant.

We first evaluated total and H-specific GDF-15 levels at baseline by combining lean participants from Study-1 and 2 (*n* = 28) in one analysis. As previously described, approximately 15–35% of the population carry a GDF-15 missense variant (H202D variant)^13^. We confirm this percentage in the population sample studied herein using ELISAs, detecting total GDF-15 in all participants. Still, H-specific was detected in only 75% of participants -suggesting that the rest 25% of the participants carried the H202D variant^13^. Participants with the H202D variant had higher total GDF-15 levels compared to participants without it (Table 1a), an observation that was more pronounced in individuals with REDs (Table 1c). However, it is unclear if the H202D variant may interfere with the bioactivity of its parent molecule, primarily under metabolic stress. A recent study demonstrated similar synthetic H-specific and GDF-15 activity with the variant in a luciferase assay with cells coated with GFRAL^13^. In agreement with this, our findings indicate no differences in the GDF-15 association with metabolites-lipids-lipoproteins at baseline between individuals with and without the H202D variant (Fig. 7a), suggesting that total and H-specific GDF-15 might have a similar biological effect, as proposed by Karusheva et al., under normally fed conditions^13^.

In contrast, during acute complete starvation, participants with the H202D variant display more robust changes in VLDLs and acetate, compared to the ones without the variant (Fig. 7b); this finding may suggest a difference in the bioactivity of total and H-specific GDF-15 during stressful conditions, like starvation, which could induce GDF-15 upregulation. This finding is in agreement with previous studies demonstrating GDF-15 bioactivity differences during inflammatory and stressful conditions wherein the variant is present^14–16,23^.

We demonstrate that GDF-15 increases significantly with acute fasting (Fig. 2). These findings are consistent with the proposed classification of GDF-15 as a stress hormone, which is thus expected to increase with starvation. In murine studies, GDF-15 levels were increased following acute short-term fasting, particularly after 24 h^27^. Along these lines, an increase of total GDF-15 with caloric restriction of various durations and intensities has been recently shown^27^. This study demonstrated that humans under complete 7-day caloric deprivation markedly increased GDF-15, which peaked at 48 h and then gradually returned to normal. In agreement with these findings, we show herein that 3-day complete fasting leads to a progressive and significant elevation of total and H-specific GDF-15. This elevation became significant compared to the control–fed state at 48 h from baseline (Fig. 2).

Despite previous animal studies suggesting GDF-15’s role in weight regulation^8,11^, our data preclude that GDF-15 is involved in body weight regulation, given that, in that case, a decrease and not an increase of circulating GDF-15 levels would have been expected in response to food deprivation. This is in concordance with recent previous studies in humans showing no association between weight regulation and GDF-15 levels^4,5^. Therefore, differences between animals’ and humans’ GDF-15 biological functions might exist.

We then explored any potential associations between the GDF-15 and the leptin axis. At baseline, a negative association was established between GDF-15 and leptin levels, which could be causal and direct or could merely reflect an underlying association with negative energy balance, which in turn is reflected by leptin levels (Fig. 2c). Thus, we corrected fasting-induced hypoleptinemia to explore whether leptin drives fasting-induced GDF-15 changes. Despite the negative association between leptin and total GDF-15 at baseline, leptin replacement did not block the GDF-15 increase in response to fasting, indicating no causal relationship. Our findings support the hypothesis that GDF-15 upregulation is independent of leptin, at least in the context of acute and long-term food deprivation (Fig. 2). Overall, we hypothesize that GDF-15 functions in humans and mice differ^1a1^, especially in regards to weight regulation and interactions with leptin, a hormone which in turn is also known to function differently in mice and humans^17^.

GDF-15 is considered a stress cytokine that is elevated in response to inflammation or mitochondrial dysfunction^28–30^. In both of our studies (short and long-term), leptin administration failed to restore GDF-15 levels to normal (Figs. 2 and 3). As a result, even though there was a negative association between GDF-15 and leptin levels, this is probably driven by the underlying energy state and not through a direct causal relationship between the two pathways. Regarding chronic mild caloric deprivation, according to our findings, females with REDs from Study-2 have significantly higher total and H-specific GDF-15 compared to their healthy lean counterparts, particularly in the presence of the H202D variable (Table 1). REDs is considered a state of prolonged body stress due to energy deficits leading to homeostasis dysregulation and neuroendocrine dysfunction^19,20^. Thus, we hypothesized that the levels of GDF-15, a stress-response cytokine, would be elevated in these participants. Furthermore, participants of Study 2 received a placebo or leptin for 36 weeks, aiming to correct the energy deficiency-induced hypoleptinemia, and thus, we sought to explore any effects of leptin on GDF-15 levels. Even though long-term leptin administration restored leptin to supraphysiological levels, it failed to affect GDF-15 (Fig. 3). This reinforces the hypothesis that leptin and GDF-15 act through independent pathways in humans, which is consistent with a study in patients with lipodystrophy and hypoleptinemia, wherein leptin treatment failed to normalize GDF-15 levels^31^.

Since the physiological role of GDF-15 remains unclear, we performed exploratory correlations of GDF-15 with metabolites, lipoproteins, and lipids (a) at baseline (Figs. 4 to 6) and (b) during changes from day 1 to day 4 in response to starvation, i.e., correlations were performed using the delta difference between the first and last day of fasting and placebo or leptin administration (Supplementary Figs. 1, 2). At baseline, total GDF-15 levels were significantly and positively correlated with triglyceride-rich particles, including VLDLs and their ratios with other lipid particles, triglyceride-rich lipoproteins, as well as glycerol (component of triglyceride synthesis) (Figs. 4 to 6). The liver is one of the main organs responsible for GDF-15 production, and VLDL is also produced and released by the liver to carry mostly triglycerides to other body tissues. In a recent study, Zhang et al. injected adenovirus expressing GDF-15 in obese mice. The injected mice demonstrated a significant reduction in liver weight, hepatocyte triglyceride content, and steatosis compared to controls^32^. The above and other similar findings suggest a strong connection between GDF-15, liver, and lipids and raise the question of whether GDF-15 is a mobilizer of liver lipids and thus could play a role not only in starvation but also in liver metabolic diseases. On the other hand, baseline total GDF-15 was positively correlated to the amino acids glycine and phenylalanine. A previous study suggested that an excess of phenylalanine could upregulate the transcription of GDF-15^33^; however, there is limited data related to GDF-15 and these amino acids.

During the evaluation of changes in fasting between day 1 and day 4, GDF-15 was positively correlated with HDL particles. A previous study incorporating Mendelian Randomization analyses showed a causal relationship between GDF-15 and HDL^34^; however, the available data is limited, and additional studies are needed to evaluate GDF-15’s role in glucolipid and cardiometabolic diseases^35^. Furthermore, with regards to fatty acids, GDF-15 was positively correlated with arachidic acid (C20:0) at baseline and with changes of arachidonic acid (C20:4n6) and α-linolenic acid (C18:3n3) during the delta changes of fasting (Fig. 5 and Supplementary Fig. 2). Arachidic acid is structurally related to arachidonic acid, which has been shown to react with various mitochondrial enzymes, inducing oxidative stress and upregulating the expression of GDF-15 as a response^36^. Further studies need to more precisely explore the association between GDF-15 and fatty acids^37^, particularly under prolonged fasting and metabolic stress.

As further aims, we sought to compare the metabolic-lipidic responses to starvation between people with and without the GDF-15 studied variant. The presented sPLS-DA differentiated between people with and without the H202D variant, especially during fasting (Fig. 7d). At the isocaloric fed state, there were no significant differences in the metabolites-lipids-lipoproteins between the two groups, suggesting that at least under normal fed conditions, there might be fewer differences in the biological role of the two molecules. In contrast, acute complete fasting resulted in significant differences in the contents of VLDLs and acetate (Fig. 7b). Even though this effect became borderline significant after FDR correction, sPLS-DA analysis demonstrated clusters of parameters (i.e., mostly VLDLs in Component-1 and HDLs in Component-3) being differentially regulated in the participants with and without the H202D variant (Fig. 7d). This raises the hypothesis that although there might be no major difference between the two groups under normal baseline-fed conditions, when the system is stressed through acute complete starvation, and GDF-15, as a stress-response cytokine is upregulated, differences between the biological role of total and H-specific GDF-15^27,32,38^ may become prominent, as demonstrated herein. Higher total GDF-15 levels were also seen in subjects with REDs (chronic metabolic stress) and the H202D variant compared to their counterparts without its presence (Table 1c). Thus, this finding should be explored in further studies with larger sample sizes, which will allow differences, if any, between individuals with and without the H202D variant to be more evident.

The possibility of systematic bias in GDF-15 measurements^13^ and whether the H202D variant alters GDF-15 levels is a main concern in the interpretation of previous studies and GDF-15’s function. Recently, a study demonstrated significant differences in GDF-15 levels measured with Roche-Elecsys ELISA and R&D-DuoSet immunoassays in the same human samples^13^. For our studies, we evaluated and used two Ansh ELISAs, total GDF-15, which detects GDF-15 levels irrespective of the presence of the H202D variant, and H-specific GDF-15 which detects only GDF-15 without the H202D variant^4,5,16,23^. These kits have been internally and externally validated and have been used in papers published by several different groups^4,5,16,22,23^. For instance, both Ansh GDF-15 kits were first validated with an established recombinant human GDF-15 peptide (Sino-Biologicals, Cat:10936-H07Y) that has previously been fully validated independently in preclinical, experimental systems^39–41^ (Supplementary Table 4b, Supplementary Fig. 6). Besides, the kits have also been externally validated with the MSD R&D DuoSet ELISA^23^, and mass spectrometry characterized synthetic recombinant GDF-15 proteins peptides, including a HH homodimeric of the H amino acid at position 202 of the unprocessed GDF-15 form, a DD homodimeric peptide of the D amino acid on the same position, and an HD^22,23^. These synthetic MS-verified protein peptides were independently developed and provided by Professor Marko Hynoven at the Department of Biochemistry at the University of Cambridge, UK, and their validation has been recently published by other independent groups^22,23^. Moreover, the manufacturer has performed additional validations for both GDF-15 Ansh ELISA kits using the same MS-verified synthetic protein peptides, as well as with the R&D calibrators (Supplementary Table 5).

We have to acknowledge several limitations. The sample size is limited, however initial sample size considerations for the trials shown herein were established on the basis of exploring the effects of leptin administration, and our current sample size is still sizeable enough to demonstrate robust changes of GDF15 and leptin. No similar studies have been published, and there were no data available to perform power calculations of leptin administration for our study, but our data can certainly serve this purpose for future studies. In comparisons between participants with and without the H202D variant, the sample size was not well-balanced; this is attributed to the fact that only 25–30% of the population carry the variant. We recognize that this difference in sample size may affect the statistical findings, and we raise herein novel hypotheses and provide initial data to be tested further in future studies. In addition, our exploratory metabolite–lipid–lipoprotein analysis did not include all circulating lipids or metabolites and did not describe lipid subgroups and individual lipid species that should be the focus of more in-depth studies in the future. Thus, this part of the study could be considered an exploratory study that raises hypotheses for future studies on GDF-15, for which modest knowledge is available. Future studies that directly administer or inhibits GDF-15 in humans should be conducted to confirm the above findings.

In summary, we are the first to evaluate total and H-specific GDF-15 in humans with acute and chronic caloric deprivation and leptin administration. We show herein that total and H-specific GDF-15 increases, as metabolic stress-related molecules do, in response to complete acute fasting and chronic mild caloric deprivation. These changes are independent of leptin, as its administration does not restore their levels to normal. In addition, our data preclude that GDF-15 is involved in body weight regulation, given that, in that case, a decrease and not an increase of circulating GDF-15 levels would have been expected. Finally, we show that total GDF-15 is positively correlated with the triglyceride-rich particles and lipoproteins, mainly VLDLs, during baseline conditions. No significant differences were detected between participants with and without the variant at baseline. Still, principal component analyses raise suggestions about possible differential effects and bioactivity of H-specific and total GDF-15 during metabolic stress that need to be tested by future, more extensive studies. Additional studies involving GDF-15 administration and/or neutralization are needed to fully elucidate whether GDF-15 is a mitokine reflecting or mediating responses to metabolic stress and caloric deprivation.

## Methods

The primary aim of our analysis was to study, in the context of randomized placebo-controlled trials, changes of total and H-specific GDF-15 in response to acute complete fasting and chronic mild caloric deficit and REDs. Secondary aims were as follows: (a) to compare baseline total and H-specific GDF-15 levels in healthy lean individuals vs. participants with REDs (previously defined as hypothalamic amenorrhea), (b) to study the changes of total and H-specific GDF-15 in response to longer-term leptin administration or placebo in lean individuals with a chronic mild caloric deficit in the context of REDs (c) to study potential differential associations of total and H-specific GDF-15 levels in lean individuals at baseline with biological sex, body composition, i.e., study outcomes that could be due to the H202D variant, (d) to correlate total and H-specific GDF-15 with components of the lipid and metabolic profile (i.e., lipoproteins, amino acids, fatty acids, ketone bodies). We utilized data and specimens from our previous studies to perform new measurements and analyses^19,26,42,43^.

### Study approval

The human studies were approved by the Institutional Review Board of Beth Israel Deaconess Medical Center and were performed at the General Clinical Research Center (GCRC) in accordance with the declaration of Helsinki under an investigator-held IND. All participants obtained written informed consent before inclusion in any of the studies.

Human Study-1: Cross-over study of acute complete fasting with placebo or leptin administration for 3 days in lean, healthy males and females

We used data and specimens from our previous studies to perform new measurements and analyses^26,43^. Six healthy lean men (mean age 23.50 ± 1.54 years) and seven healthy lean women (mean age 23.71 ± 1.46 years) with regular menstrual cycles and not on oral contraceptives for at least 6 months were studied under three separate GCRC-based conditions for 72 h: one under isocaloric fed state conditions and two during complete fasting state conditions scheduled in random order and in a double-blind fashion with the administration of placebo (fasting + placebo) or physiologic replacement leptin doses (fasting + leptin). Inclusion criteria were determined for healthy lean participants. Exclusion criteria included a history of any illness that may affect the concentrations of the hormones to be studied, medications known to affect the hormones to be measured, history of anaphylactoid-like reactions, or known hypersensitivity to E. coli-derived proteins. Participants were free-living between the 8 weeks interval between admissions, allowing participants to recover body weight, leptin levels, and hematocrit. Each participant completed three studies (i.e., fed, fasting + placebo, fasting + leptin), with the exception of one female who did not complete the fasting + placebo study. During each fed or fasting study, participants were admitted to the GCRC the evening before study day 1. The isocaloric fed state consisted of four standardized meals per day: breakfast (20% of daily calories) at 8:00, lunch (35% of daily calories) at 13:00, dinner (35% of daily calories) at 18:00 and a snack (10% of daily calories) at 22:00. During the fasting state, only a standardized volume of calorie-free fluids, electrolytes (NaCl [500 mg] and KCL [40 meq], and vitamin supplements were allowed. Body composition (bioelectric impedance analysis; RJL Systems, Clinton Township, MI), RMR (DeltaTrac II Metabolic Monitor; SensorMedics), and morning vital signs were assessed at the beginning and end of each study. The doses of leptin were 0.01 mg/kg given at 8:00 and every 6 h on day 1, 0.025 mg/kg at 8:00 and every 6 h on days 2 and 3 for males, and 0.02 mg/kg given at 8 am and every 6 h on day 1 and 0.05 mg/kg given at 8 am and every 6 h on days 2 and 3 for females (Amylin, Inc., San Diego, CA; previously known as r-metHuLeptin, provided by Amgen, Inc., Thousand Oaks, CA) administrated subcutaneously. Males and females were administered a single dose of 0.025 mg/kg and 0.05 mg/kg, respectively, at 8:00 on day 4. Blood samples obtained at 8:00-8:30 on days 1 and 4 were used for our current measurements and analysis^26,43^. [ClinicalTrials.gov Study-1: NCT00140231].

Human Study-2: Randomized control trial of long-term leptin administration vs. placebo administration for 36 weeks in females with chronic mild energy deficiency.

We utilized data and specimens from our previous studies to perform new measurements and analyses^19^. Fifteen females between 18 and 35 years old with REDs (manifesting and previously being specified as hypothalamic amenorrhea per protocol inclusion criteria) for ≥6 months coincident with strenuous exercise and/or low body weight (within ±15% of ideal body weight for ≥6 months at the time of screening), were studied^19,24^. All participants were otherwise healthy, without active eating disorders or other psychiatric diseases, and were not taking any medications that could affect hormone or bone mass measurements (i.e., glucocorticoids, antiseizure medications, thyroid hormones, or estrogens) for at least 3 months. Exclusion criteria included patients with significant medical history or other endocrine causes of amenorrhea or pregnancy; patients with alcoholism, drug abuse, smoking history, active eating disorders, depression, or other psychiatric diseases, or participants on medications known to affect the hormones to be measured. None of the participants had hyperprolactinemia, hypo- or hyperthyroidism, Cushing’s syndrome, congenital adrenal hyperplasia, or primary ovarian failure. Participants were randomized to receive either metreleptin or placebo for 36 weeks. Randomization tables were produced by the Harvard Catalyst biostatisticians with SAS and delivered directly to the Research Pharmacy for use of the study staff that recruited participants, as well as the participants, who remain blinded. Metreleptin was self-injected subcutaneously once daily at a dose of 0.08 mg/kg/day for 12 weeks, and participants who had begun menstruating remained on this dose until the completion of the study. The dose for participants who had not menstruated at week 12 was increased to 0.12 mg/kg/day. If a participant lost >5% of her baseline weight, the dose was reduced by 0.04 mg/kg. Vital signs and body weight measurements were performed every 4 weeks. Body composition and RMR were measured every 12 weeks with DEXA and Sensormedics Vmax Encore equipment (VIASYS Respiratory Care Inc,), respectively. Fasting blood samples from the participants (leptin-treated = 9 and placebo-treated = 6) obtained at weeks 0, 12, 24, and 36 of the study were used for the current measurements^19^. [ClinicalTrials.gov Study-2: NCT00130117].

### Biochemical analyses

Total GDF-15 and H-specific (H202D non-detectable) GDF-15 were measured using the available Ansh Labs LLC (Webster|TX | USA) ELISAs^4,5,16,22^. All serum samples were run in duplicate within the same run for a given participant and repeated if any sample’s coefficient of variation (CV) was >15%. The GDF-15 ELISAs were performed in the Mantzoros Laboratory, Beth Israel Deaconess Medical Center, Boston, MA, using the Accuris™ SmartReader™ 96 microplate absorbency reader model MR9600-T (Edison|NJ | US). All technical characteristics of other hormones used in our analysis have been previously measured and published^19,42^.

### Precision, linearity, and sensitivity of the GDF-15 ELISAs

We initially assessed the assays’ precision internally using two concentrations of controls in each plate, one at the low range and one at the high range of the assay, for a total of 35 times per control measured in the same run (Supplementary Table 4a). The linearity of the assay was calculated with dilutions of an initial concentration of 2600 pg/mL, and the percentages of recovery are shown in (Supplementary Table 4d). Furthermore, the analytical sensitivities of the assays were internally calculated by the interpolation of mean plus two standard deviations of 16 replicates of calibrator A (0 pg/mL). The calculated sensitivities are 5.83 pg/mL for total GDF-15 and 13.24 pg/mL for H-specific GDF-15.

### Total and H-specific Ansh GDF-15 ELISAs external, independent validation

External validations for both total and H-specific GDF-15 Ansh ELISA kits were performed in independent research groups with different methodologies. First, the validation of the assays was performed with a known, fully validated, and previously published GDF-15 peptide from the Human Recombinant Protein (Cat: 10936-H07Y) Sino Biologicals, which consists of 122 amino acids, and it predicts a molecular mass of 13.7 kDa^39–41^ in the Kelesidis Laboratory, UCLA, Los Angeles, CA. For both total and H-specific GDF-15 kits, the percentage recovery for the expected GDF-15 value was 100%+/− 5%. These evaluations were done in buffer and plasma; however, for Supplementary Table 4b and Supplementary Fig. 6, plasma is shown since this is the actual matrix used in our study herein. The assays’ precision was performed by estimating the intra-assay CV of 8 serial dilutions of a specific peptide concentration using 3 technical replicates for each dilution. For both total and H-specific GDF-15 assays, intraassay CVs (between runs) were lower or equal to 13.58%, thus within an acceptable range (CV < 15%) (Supplementary Table 4b).

The accuracy of the Ansh GDF-15 ELISA has also been validated with recombinant preparations of mass spectrometry verified, synthetic GDF-15 peptides, including the wild-type (HH, homodimeric) and DD variants in the 202 positions of the unprocessed GDF-15 form, as well as HD variants, tested by independent groups^22,23^. These recombinant synthetic proteins were created and provided by the Department of Biochemistry at the University of Cambridge, UK, and the detailed validation was recently published^22,23^. To highlight, both independent groups and the manufacturer validated the Ansh kit with calibrators and against the R&D DuoSet ELISA^22,23^. The calibrators from the Ansh Labs ELISA are traceable to the recombinant human R&D Systems (Biotechne, USA) GDF‐15^23^. In addition, analytes, percentages of reactivity, sensitivity, and precision have also been previously published by independent groups^16^. Finally, Ansh Labs GDF-15 kits have been previously used and published in previous studies^4,5,16,23^. On the other hand, the manufacturer also utilized these synthetic mass spectrometry-verified GDF-15 proteins with a concentration of 50 pg/mL in a study using controls and one serum pool. The study included a total of 6 assays, three replicates of each per assay (*n* = 18). The total GDF-15 ELISA detects both recombinant preparations equally. The H-specific assay detects HH at 100% and does not detect any D variant. This and other major technical characteristics of Ansh’s total and H-specific GDF-15 ELISAs are publicly available on their webpage and summarized in Supplementary Table 5.

### Lipoproteins, lipids, and metabolites measurements

Metabolites and lipids were initially measured with NMR spectroscopy by Nightingale Health (Helsinki, Finland), and serum fatty acids were measured with Gas Chromatography–Mass Spectrometry^24^. The complete list of the biomarkers measured with this method and used for the first analysis can be found in Supplementary Table 2a. To further confirm these results, we also performed lipoproteins and metabolites analysis measured with Nuclear magnetic resonance spectroscopy (NMR) analysis by Labcorp (Morrisville, USA). A complete list and report of their analysis can be found in Supplementary Table 2b. NMR spectra were acquired on a Vantera® Clinical Analyzer, a 400 MHz NMR instrument, from EDTA plasma samples as described for the NMR LipoProfile® test (Labcorp, Morrisville, NC)^44^.

### Statistical analysis

Statistical analysis was performed with SPSS v 28.0.1.0 (SPSS, Inc, Chicago, IL) for Windows, with GraphPad Prism 9.3.1 (GraphPad Software Inc, La Jolla, CA), R Studio (GGally package), and with MetaboAnalyst R. No a priori power calculations were performed for the current analysis, but the sample sizes were appropriate to detect differences in circulating leptin concentrations across both studies, whereas sample size considerations have been previously discussed in detail^24^. The primary outcome for our current analysis was total GDF-15 levels, and H-specific, as well as other measures, were secondary and exploratory. Results are presented in figures and tables as mean ± standard errors of the mean (SEM). Across all applicable figures, individual data points are also shown. For comparisons of participant baseline characteristics, both independent sample *T*-test and ANCOVA were performed (Table 1), as well as Mann Whitney U test, without significant differences between parametric and non-parametric tests (Supplementary Table 1). Although a non-parametric test would be more appropriate due to the distribution of the variables, given the total number of participants.

To analyze the changes in total and H-specific GDF-15 with Study-1 and Study-2, we fitted mixed effects models adjusted for baseline for both studies. Compound symmetry was used as the repeated covariance type. For Study-1, participants were matched by time (Days 1–4) and intervention, given the design of the study as a randomized cross-over trial. We set Days (1–4) and Intervention (i.e., fed, fasting treated with placebo, and fasting treated with leptin) as well as the day*intervention interaction as fixed effects. For Study-2, fixed effects likewise included Time(weeks) (corresponded to weeks 0, 12, 24, and 36 of the study), Intervention (i.e., placebo and leptin treatment), and the time*intervention interaction. For between-group and between-timepoint comparisons, Fisher’s Least Significant Difference (LSD) post-hoc tests were performed between the total means of the groups, and between the means of the groups in the individual time points when the two-factor interaction (i.e., Days(1–4)*Intervention for Study-1 and Time(weeks)*Intervention for Study-2) *p*-values were <0.05. For the simple longitudinal comparison of total and H-specific GDF-15 in Study-1, a paired *t*-test was used to compare different days within each intervention.

For exploratory correlations analysis with applicable metabolo-lipidomics, data were log-transformed to ensure uniform distribution, and Pearson parametric correlation tests and partial correlations adjusting for covariates (presence of the H202D variant) were implemented, presented both before and following adjustment for the FDR per the Benjamini–Hochberg correction. To extrapolate physiological insights from our exploratory correlations across all studies and increase sample size, following log-transformation, the average of baseline values for the three interventions (i.e., fed, fasting+placebo, fasting + leptin) in Study-1 and for baseline in Study-2 were used. In Study-1, deltas (Δ) were calculated as the difference between day 1 and day 4 of each fasting intervention (i.e., fasting+placebo, fasting+leptin).

For the metabolomics analysis, missing values were imputed with the 1/5 of the minimum value. We plotted the results of t-tests comparing deltas of metabolites in people with and without the H202D variant at baseline and after 3 days, corrected for FDR. A sPLS-DA was also performed to reduce data dimensionality and classify metabolite-lipoprotein-lipid profiles between participants with and without the H202D variant. First, we performed this analysis using only baseline values in Study-1 and Study-2. We then repeated the same analysis using delta changes only for Study-1.

Our study is a small single‐center study, which was powered and showed significant results for the primary outcome. However, it was not powered to detect effect sizes with adjustment for multiple comparisons in the exploratory analysis for the H202D variant as per standard practice. Instead, consistency, direction, and magnitude of the effect in conjunction with the nominal P values were considered to help distinguish true‐ and false‐positive findings^4,5^. A subsequent study with preplanned hypotheses should be conducted to confirm the observed associations.

### Additional resources

Clinical trial registry numbers are the following: ClinicalTrials.gov Study-1: NCT00140231; ClinicalTrials.gov Study-2: NCT00130117 available in ClinicalTrials.gov.

### Reporting summary

Further information on research design is available in the Nature Portfolio Reporting Summary linked to this article.

### Supplementary information

Supplementary Information

Reporting Summary

### Source data

Source Data

## Data Availability

All data supporting the findings described in this manuscript are available in the article and the Supplementary Information, and from the corresponding author upon request. Relevant information on the ELISA assay used is available in Supplementary Tables 4 and 5 and Supplementary Fig. 6. A list of all identified metabolites is available in Supplementary Table 2. Source data are provided with this paper. Data is subject to controlled access and is not publicly accessible owing to institutional regulations but will be made available from the corresponding author upon request. A signed data transfer agreement between the corresponding author’s institution and the requesting institution is required. The timeframe for responses to relevant requests will be immediate (a few days). Standard legal restrictions per data transfer agreements will apply.

## Code availability

Applicable code supporting the results presented herein is available from the corresponding author upon reasonable request.
