# Supplementary material for: Total and H-specific GDF-15 levels increase in caloric deprivation independently of leptin in humans
Source: Nat Commun. 2024 Jun 18;15:5190. doi: 10.1038/s41467-024-49366-y (PMC11189399; doi:10.1038/s41467-024-49366-y)
Supplement: Supplementary file 1 — Supplementary Information [file 41467_2024_49366_MOESM1_ESM.pdf]

Supplementary Table-1:

a. Baseline characteristics with non-parametric tests for Study-1 and 2.

Variables	Men (n=6)	Women (n=22)	p-value[a]	Without the H202D variant (n=21)	With the H202D variant (n=7)	p-value[b]
Age (Years)	22 (7)	23 (7)	0.336	23 (7)	22 (8)	**0.043**
Weight (Kg)	78.95 (9.99)	55.65 (14.51)	**<0.001**	61.79 (22.77)	54.28 (9.42)	0.150
Fat mass (Kg)	12.40 (4.03)	14.78 (5.10)	0.283	14.10 (5.14)	12.19 (7.48)	0.318
Lean mass (Kg)	64.20 (8.07)	41.54 (10.67)	**<0.001**	43.35 (21.70)	40.98 (8.98)	0.218
BMR (Kcal/day)	1670.96 (361.96)	1260.00 (243)	**<0.001**	1305 (511.18)	1195 (210)	0.165
Total GDF-15 (pg/mL)	556.60 (103.96)	529.46 (285.08)	0.365	549.59 (144.27)	704.26 (755.09)	**0.018**
H-specific GDF-15 (pg/mL)	428.26 (312.34)	337.18 (234.60)	1.000	344.43 (272.95)	NA	NA
HR (BPM)	54.83 (7.71)	58 (14.34)	0.935	56.84 (11.58)	57.67 (17.33)	0.862
SBP (mmHg)	120.84 (20.71)	101 (14.50)	**<0.001**	105.55 (16.54)	101.33 (23)	0.328
DBP (mmHg)	67.67 (9.54)	59 (11.67)	**<0.001**	63.17 (13.46)	58 (14)	0.328
Leptin (ng/mL)	1.93 (4.28)	7.5 (8.47)	**0.017**	4..92 (9.48)	4.86 (4.25)	0.901
FFA (mmol/L)	0.46 (0.09)	0.23 (0.57)	0.110	0.34 (0.45)	0.29 (0.41)	0.725
Cortisol (µg/dL)	11.70 (1.88)	18.90 (5.30)	**<0.001**	17.80 (22.77)	16.33 (9.24)	1.000
Aldosterone (pg/mL)	81.67 (48.41)	60.99 (48.23)	0.836	69 (44.64)	78.41 (41.99)	0.724
Free T4 (ng/dL)	0.83 (0.35)	1.07 (0.38)	0.126	0.99 (0.39)	0.85 (0.65)	0.823

Data are presented as median and interquartile ranges (IQR). Two-sided p-values are presented, with significance (<0.050) indicated in bold.
[a]: Whitney U test between males (n=6) and females (n=22).
[b]: Whitney U test between subjects with (n=7) and without the H202D variant (n=21).
Abbreviation list: BMR, body metabolic rate; BPM, beats per minute; DBP, Diastolic blood pressure; FFA, Free fatty acids; GDF-15, Growth differentiation factor-15; HR, Heart rate; SBP, Systolic blood pressure; T4, Thyroxine.

b. Baseline characteristics with non-parametric tests for Study-1.

Variables	Men (n=6)	Women (n=7)	p-value[a]	Without the H202D variant (n=10)	With the H202D variant (n=3)	p-value[b]
Age (Years)	22 (7)	22 (9)	1.000	22 (8)	24	0.371
Weight (Kg)	78.95 (9.99)	55.65 (13.45)	**0.005**	69.61 (9.99)	55.78	0.287
Fat mass (Kg)	12.40 (4.03)	16.10 (6.96)	**0.035**	14.10 (5.98)	13.30	0.573
Lean mass (Kg)	64.20 (8.07)	40.98 (6.42)	**0.001**	52.69 (26.26)	40.98	0.371
BMR (Kcal/day)	1670.96 (361.96)	1260.00 (153.33)	**0.002**	1559.59 (451.67)	1253.33	0.161

**Total GDF-15 (pg/mL)**	556.60 (103.96)	484.61 (225.86)	0.138	498.96 (185.73)	567.18	0.217
**H-specific GDF-15 (pg/mL)**	428.26 (312.34)	337.18 (311.08)	0.690	357.53 (308.43)	NA	NA
**HR (BPM)**	54.83 (7.71)	61.33 (15.33)	**0.008**	58.25 (1.40)	64.02	0.937
**SBP (mmHg)**	120.84 (20.71)	101.33 (8.33)	**0.005**	112.25 (25.59)	107	0.811
**DBP (mmHg)**	67.67 (9.54)	61.67 (8.33)	**0.005**	65.41 (6.92)	61.67	0.692
**Leptin (ng/mL)**	1.93 (4.28)	12.64 (4.07)	**0.001**	8.45 (11.54)	6.75	0.692
**FFA (mmol/L)**	0.46 (0.09)	0.06 (0.03)	**0.001**	0.18 (0.42)	0.07	0.573
**Cortisol (µg/dL)**	11.70 (1.88)	16.57 (3.25)	**0.014**	14.50 (6.71)	12.76	0.692
**Aldosterone (pg/mL)**	81.67 (48.41)	53.59 (30.64)	0.534	65.30 (42.73)	71.95	0.937
**Free T4 (ng/dL)**	0.83 (0.35)	1.23 (0.36)	**0.035**	0.93 (0.35)	1.41	**0.014**

Data are presented as median and interquartile ranges (IQR). Two-sided p-values are presented, with significance (<0.050) indicated in bold.
[a]: Whitney U test between males (n=6) and females (n=7).
[b]: Whitney U test between subjects with (n=3) and without the H202D variant (n=10).
Abbreviation list: BMR, body metabolic rate; BPM, beats per minute; DBP, Diastolic blood pressure; FFA, Free fatty acids; GDF-15, Growth differentiation factor-15; HR, Heart rate; SBP, Systolic blood pressure; T4, Thyroxine.

c. Baseline characteristics with non-parametric tests for Study-2.

Variables	Without the H202D variant (n=11)	With the H202D variant (n=4)	p-value
**Age (Years)**	26 (5)	29 (10)	0.099
**Weight (Kg)**	55.10 (14.64)	52.37 (13.13)	0.440
**Fat mass (Kg)**	13.86 (5.23)	11.22 (6.76)	0.679
**Lean mass (Kg)**	42 (12.62)	40.98 (8.98)	0.371
**BMR (Kcal/day)**	1138.50 (265.25)	1169 (260.50)	0.513
**Total GDF-15 (pg/mL)**	580.64 (357.19)	1276.85 (758.94)	**0.013**
**H-specific GDF-15 (pg/mL)**	333.16 (198.49)	NA	NA
**HR (BPM)**	51.50 (13.75)	48 (26)	1.000
**SBP (mmHg)**	99 (15)	90.50 (29.50)	0.310
**DBP (mmHg)**	57.50 (12.25)	54 (14)	0.513
**Leptin (ng/mL)**	4.04 (4.95)	2.84 (3.42)	0.440
**FFA (mmol/L)**	0.48 (0.53)	0.31 (0.28)	0.304
**Cortisol (µg/dL)**	21.60 (5.63)	19.60 (7.57)	1.000
**Aldosterone (pg/mL)**	73.53 (50.94)	85.32 (100.86)	0.943

Free T4 (ng/dL)	1.05 (0.64)	0.76 (0.21)	0.055

Data are presented as median and interquartile ranges (IQR). Two-sided p-values are presented, with significance (<0.050) indicated in bold.
p-value: Whitney U test between subjects with (n=4) and without the H202D variant (n=11).
Abbreviation list: BMR, body metabolic rate; BPM, beats per minute; DBP, Diastolic blood pressure; FFA, Free fatty acids; GDF-15, Growth differentiation factor-15; HR, Heart rate; SBP, Systolic blood pressure; T4, Thyroxine.

Supplementary Table 2.

a. List of Nightingale lipids and metabolites for exploratory correlations

**Abbreviation**	**Full name**
VLDL-D	Mean diameter for very low density lipoprotein (VLDL) particles
XXL-VLDL-P	Concentration of chylomicrons and extremely large VLDL particles
XXL-VLDL-L	Total lipids in chylomicrons and extremely large VLDL
XXL-VLDL-PL	Phospholipids in chylomicrons and extremely large VLDL
XXL-VLDL-C	Total cholesterol in chylomicrons and extremely large VLDL
XXL-VLDL-CE	Cholesterol esters in chylomicrons and extremely large VLDL
XXL-VLDL-FC	Free cholesterol in chylomicrons and extremely large VLDL
XXL-VLDL-TG	Triglycerides in chylomicrons and extremely large VLDL
XL-VLDL-P	Concentration of very large VLDL particles
XL-VLDL-L	Total lipids in very large VLDL
XL-VLDL-PL	Phospholipids in very large VLDL
XL-VLDL-C	Total cholesterol in very large VLDL
XL-VLDL-CE	Cholesterol esters in very large VLDL
XL-VLDL-FC	Free cholesterol in very large VLDL
XL-VLDL-TG	Triglycerides in very large VLDL
L-VLDL-P	Concentration of large VLDL particles
L-VLDL-L	Total lipids in large VLDL
L-VLDL-PL	Phospholipids in large VLDL
L-VLDL-C	Total cholesterol in large VLDL
L-VLDL-CE	Cholesterol esters in large VLDL
L-VLDL-FC	Free cholesterol in large VLDL
L-VLDL-TG	Triglycerides in large VLDL
M-VLDL-P	Concentration of medium VLDL particles
M-VLDL-L	Total lipids in medium VLDL
M-VLDL-PL	Phospholipids in medium VLDL
M-VLDL-C	Total cholesterol in medium VLDL
M-VLDL-CE	Cholesterol esters in medium VLDL
M-VLDL-FC	Free cholesterol in medium VLDL

M-VLDL-TG	Triglycerides in medium VLDL
S-VLDL-P	Concentration of small VLDL particles
S-VLDL-L	Total lipids in small VLDL
S-VLDL-PL	Phospholipids in small VLDL
S-VLDL-C	Total cholesterol in small VLDL
S-VLDL-CE	Cholesterol esters in small VLDL
S-VLDL-FC	Free cholesterol in small VLDL
S-VLDL-TG	Triglycerides in small VLDL
XS-VLDL-P	Concentration of very small VLDL particles
XS-VLDL-L	Total lipids in very small VLDL
XS-VLDL-PL	Phospholipids in very small VLDL
XS-VLDL-C	Total cholesterol in very small VLDL
XS-VLDL-CE	Cholesterol esters in very small VLDL
XS-VLDL-FC	Free cholesterol in very small VLDL
XS-VLDL-TG	Triglycerides in very small VLDL
XXL-VLDL-PL_%	Phospholipids to total lipids ratio in chylomicrons and extremely large VLDL
XXL-VLDL-C_%	Total cholesterol to total lipids ratio in chylomicrons and extremely large VLDL
XXL-VLDL-CE_%	Cholesterol esters to total lipids ratio in chylomicrons and extremely large VLDL
XXL-VLDL-FC_%	Free cholesterol to total lipids ratio in chylomicrons and extremely large VLDL
XXL-VLDL-TG_%	Triglycerides to total lipids ratio in chylomicrons and extremely large VLDL
XL-VLDL-PL_%	Phospholipids to total lipids ratio in very large VLDL
XL-VLDL-C_%	Total cholesterol to total lipids ratio in very large VLDL
XL-VLDL-CE_%	Cholesterol esters to total lipids ratio in very large VLDL
XL-VLDL-FC_%	Free cholesterol to total lipids ratio in very large VLDL
XL-VLDL-TG_%	Triglycerides to total lipids ratio in very large VLDL
L-VLDL-PL_%	Phospholipids to total lipids ratio in large VLDL
L-VLDL-C_%	Total cholesterol to total lipids ratio in large VLDL
L-VLDL-CE_%	Cholesterol esters to total lipids ratio in large VLDL
L-VLDL-FC_%	Free cholesterol to total lipids ratio in large VLDL
L-VLDL-TG_%	Triglycerides to total lipids ratio in large VLDL
M-VLDL-PL_%	Phospholipids to total lipids ratio in medium VLDL
M-VLDL-C_%	Total cholesterol to total lipids ratio in medium VLDL
M-VLDL-CE_%	Cholesterol esters to total lipids ratio in medium VLDL
M-VLDL-FC_%	Free cholesterol to total lipids ratio in medium VLDL
M-VLDL-TG_%	Triglycerides to total lipids ratio in medium VLDL

S-VLDL-PL_%	Phospholipids to total lipids ratio in small VLDL
S-VLDL-C_%	Total cholesterol to total lipids ratio in small VLDL
S-VLDL-CE_%	Cholesterol esters to total lipids ratio in small VLDL
S-VLDL-FC_%	Free cholesterol to total lipids ratio in small VLDL
S-VLDL-TG_%	Triglycerides to total lipids ratio in small VLDL
XS-VLDL-PL_%	Phospholipids to total lipids ratio in very small VLDL
XS-VLDL-C_%	Total cholesterol to total lipids ratio in very small VLDL
XS-VLDL-CE_%	Cholesterol esters to total lipids ratio in very small VLDL
XS-VLDL-FC_%	Free cholesterol to total lipids ratio in very small VLDL
XS-VLDL-TG_%	Triglycerides to total lipids ratio in very small VLDL
IDL-P	Concentration of IDL particles
IDL-L	Total lipids in intermediate density lipoproteins (IDL)
IDL-PL	Phospholipids in IDL
IDL-C	Total cholesterol in IDL
IDL-CE	Cholesterol esters in IDL
IDL-FC	Free cholesterol in IDL
IDL-TG	Triglycerides in IDL
IDL-PL_%	Phospholipids to total lipids ratio in IDL
IDL-C_%	Total cholesterol to total lipids ratio in IDL
IDL-CE_%	Cholesterol esters to total lipids ratio in IDL
IDL-FC_%	Free cholesterol to total lipids ratio in IDL
IDL-TG_%	Triglycerides to total lipids ratio in IDL
LDL-D	Mean diameter for low density lipoprotein (LDL) particles
L-LDL-P	Concentration of large LDL particles
L-LDL-L	Total lipids in large LDL
L-LDL-PL	Phospholipids in large LDL
L-LDL-C	Total cholesterol in large LDL
L-LDL-CE	Cholesterol esters in large LDL
L-LDL-FC	Free cholesterol in large LDL
L-LDL-TG	Triglycerides in large LDL
M-LDL-P	Concentration of medium LDL particles
M-LDL-L	Total lipids in medium LDL
M-LDL-PL	Phospholipids in medium LDL
M-LDL-C	Total cholesterol in medium LDL
M-LDL-CE	Cholesterol esters in medium LDL
M-LDL-FC	Free cholesterol in medium LDL

M-LDL-TG	Triglycerides in medium LDL
S-LDL-P	Concentration of small LDL particles
S-LDL-L	Total lipids in small LDL
S-LDL-PL	Phospholipids in small LDL
S-LDL-C	Total cholesterol in small LDL
S-LDL-CE	Cholesterol esters in small LDL
S-LDL-FC	Free cholesterol in small LDL
S-LDL-TG	Triglycerides in small LDL
L-LDL-PL_%	Phospholipids to total lipids ratio in large LDL
L-LDL-C_%	Total cholesterol to total lipids ratio in large LDL
L-LDL-CE_%	Cholesterol esters to total lipids ratio in large LDL
L-LDL-FC_%	Free cholesterol to total lipids ratio in large LDL
L-LDL-TG_%	Triglycerides to total lipids ratio in large LDL
M-LDL-PL_%	Phospholipids to total lipids ratio in medium LDL
M-LDL-C_%	Total cholesterol to total lipids ratio in medium LDL
M-LDL-CE_%	Cholesterol esters to total lipids ratio in medium LDL
M-LDL-FC_%	Free cholesterol to total lipids ratio in medium LDL
M-LDL-TG_%	Triglycerides to total lipids ratio in medium LDL
S-LDL-PL_%	Phospholipids to total lipids ratio in small LDL
S-LDL-C_%	Total cholesterol to total lipids ratio in small LDL
S-LDL-CE_%	Cholesterol esters to total lipids ratio in small LDL
S-LDL-FC_%	Free cholesterol to total lipids ratio in small LDL
S-LDL-TG_%	Triglycerides to total lipids ratio in small LDL
HDL-D	Mean diameter for high density lipoprotein (HDL) particles
XL-HDL-P	Concentration of very large HDL particles
XL-HDL-L	Total lipids in very large HDL
XL-HDL-PL	Phospholipids in very large HDL
XL-HDL-C	Total cholesterol in very large HDL
XL-HDL-CE	Cholesterol esters in very large HDL
XL-HDL-FC	Free cholesterol in very large HDL
XL-HDL-TG	Triglycerides in very large HDL
L-HDL-P	Concentration of large HDL particles
L-HDL-L	Total lipids in large HDL
L-HDL-PL	Phospholipids in large HDL
L-HDL-C	Total cholesterol in large HDL
L-HDL-CE	Cholesterol esters in large HDL

L-HDL-FC	Free cholesterol in large HDL
L-HDL-TG	Triglycerides in large HDL
M-HDL-P	Concentration of medium HDL particles
M-HDL-L	Total lipids in medium HDL
M-HDL-PL	Phospholipids in medium HDL
M-HDL-C	Total cholesterol in medium HDL
M-HDL-CE	Cholesterol esters in medium HDL
M-HDL-FC	Free cholesterol in medium HDL
M-HDL-TG	Triglycerides in medium HDL
S-HDL-P	Concentration of small HDL particles
S-HDL-L	Total lipids in small HDL
S-HDL-PL	Phospholipids in small HDL
S-HDL-C	Total cholesterol in small HDL
S-HDL-CE	Cholesterol esters in small HDL
S-HDL-FC	Free cholesterol in small HDL
S-HDL-TG	Triglycerides in small HDL
XL-HDL-PL_%	Phospholipids to total lipids ratio in very large HDL
XL-HDL-C_%	Total cholesterol to total lipids ratio in very large HDL
XL-HDL-CE_%	Cholesterol esters to total lipids ratio in very large HDL
XL-HDL-FC_%	Free cholesterol to total lipids ratio in very large HDL
XL-HDL-TG_%	Triglycerides to total lipids ratio in very large HDL
L-HDL-PL_%	Phospholipids to total lipids ratio in large HDL
L-HDL-C_%	Total cholesterol to total lipids ratio in large HDL
L-HDL-CE_%	Cholesterol esters to total lipids ratio in large HDL
L-HDL-FC_%	Free cholesterol to total lipids ratio in large HDL
L-HDL-TG_%	Triglycerides to total lipids ratio in large HDL
M-HDL-PL_%	Phospholipids to total lipids ratio in medium HDL
M-HDL-C_%	Total cholesterol to total lipids ratio in medium HDL
M-HDL-CE_%	Cholesterol esters to total lipids ratio in medium HDL
M-HDL-FC_%	Free cholesterol to total lipids ratio in medium HDL
M-HDL-TG_%	Triglycerides to total lipids ratio in medium HDL
S-HDL-PL_%	Phospholipids to total lipids ratio in small HDL
S-HDL-C_%	Total cholesterol to total lipids ratio in small HDL
S-HDL-CE_%	Cholesterol esters to total lipids ratio in small HDL
S-HDL-FC_%	Free cholesterol to total lipids ratio in small HDL
S-HDL-TG_%	Triglycerides to total lipids ratio in small HDL

Serum-C	Serum total cholesterol
VLDL-C	Total cholesterol in VLDL
Remnant-C	Remnant cholesterol (non-HDL, non-LDL -cholesterol)
LDL-C	Total cholesterol in LDL
HDL-C	Total cholesterol in HDL
HDL2-C	Total cholesterol in HDL2
HDL3-C	Total cholesterol in HDL3
EstC	Esterified cholesterol
FreeC	Free cholesterol
Serum-TG	Serum total triglycerides
VLDL-TG	Triglycerides in VLDL
LDL-TG	Triglycerides in LDL
HDL-TG	Triglycerides in HDL
TotPG	Total phosphoglycerides
TG/PG	Ratio of triglycerides to phosphoglycerides
TotCho	Total cholines
ApoA1	Apolipoprotein A-I
ApoB	Apolipoprotein B
ApoB/ApoA1	Ratio of apolipoprotein B to apolipoprotein A-I
Glc	Glucose
Lac	Lactate
Cit	Citrate
Glol	Glycerol
Ala	Alanine
Gln	Glutamine
Gly	Glycine
His	Histidine
Ile	Isoleucine
Leu	Leucine
Val	Valine
Phe	Phenylalanine
Tyr	Tyrosine
Ace	Acetate
AcAce	Acetoacetate
bOHBut	3-hydroxybutyrate
Crea	Creatinine

Alb	Albumin
Gp	Glycoprotein acetyls, mainly a1-acid glycoprotein
PC	Phosphatidylcholine and other cholines
SM	Sphingomyelins
C14:0	Myristic acid
C16:0	Palmitic acid
C16:1	Palmitoleic acid
C18:0	Stearic acid
C18:1n9cis	Oleic acid
C18:2n6cis	Linoleic acid
C18:3n3	α-linolenic acid
C20:0	Arachidic acid
C20:1n9	Cetoleic acid
C20:2n6	Eicosadienoic acid
C20:3n6	Dihomo-gamma-linolenic acid
C20:4n6	Arachidonic acid
C22:0	Behenic acid
C20:5n3	Eicosapentaenoic acid
C22:6n3	Docosahexaenoic acid
Total FA	Total fatty acids
SFA	Saturated fatty acids
MUFA	Monounsaturated fatty acids; 16:1, 18:1
PUFA	Polyunsaturated fatty acids

High-throughput proton NMR targeted metabolomics approach (Nightingale Health Ltd, Helsinki, Finland) was used to quantify circulating metabolites and lipids within lipoprotein particles. The method leads to the simultaneous quantification of lipoprotein subclasses with lipid concentrations, fatty acids, amino acids, ketone bodies, and metabolites related to gluconeogenesis ([1]).

b. List of Labcorp lipids and metabolites for exploratory correlations

Abbreviation	Full name
TRLP	Triglyceride rich lipoprotein (TRL) particles (Total chylomicron & VLDL particles)
VL_TRLP	Very large TRL particles
L_TRLP	Large TRL particles
M_TRLP	Medium TRL particles
S_TRLP	Small TRL particles
VS_TRLP	Very small TRL particles
LDLP	Total low density lipoprotein (LDL) particles
L_LDLP	Large LDL particles
M_LDLP	Medium LDL particles

Abbreviation	Full name
S_LDLP	Small LDL particles
HDLP	Total high density lipoprotein (HDL) particles
L_HDLP	Large HDL particles
M_HDLP	Medium HDL particles
S_HDLP	Small HDL particles
H7P	H7 subspecies of HDL
H6P	H6 subspecies of HDL
H5P	H5 subspecies of HDL
H4P	H4 subspecies of HDL
H3P	H3 subspecies of HDL
H2P	H2 subspecies of HDL
H1P	H1 subspecies of HDL
TRLZ	Mean TRL size
LDLZ	Mean LDL size
HDLZ	Mean HDL size
TG	Total TRL
TC	Total Cholesterol
TRLTG	TRL triglycerides
TRLC	TRL Cholesterol
LDLC	LDL Cholesterol
HDLC	HDL Cholesterol
ApoB	Apolipoprotein B
ApoAI	Apolipoprotein A-I
BCAA	Total branched chain amino acids
Val	Valine
Leu	Leucine
Ileu	Isoleucine
Ala	Alanine
Mg	Magnesium
Ct	Citrate
KetBod	Total ketone bodies
B-HB	β-hydroxybutyrate
a-HB	a-hydroxybutyrate
AcAc	Acetoacetate
Acetone	Acetone
Glu	Glucose
GlycA	GlycA
TMAO	Trimethylamine N-oxide
Betaine	Betaine

Abbreviation	Full name
Choline	Choline
Lactate	Lactate
Pyruvate	Pyruvate
Glycine	Glycine
IVX	Inflammatory index
MMX	Metabolic malnutrition index
MVX	Metabolic vulnerability index
Creatine	Creatine
Creatinine	Creatinine
eGFR	Estimated glomerular filtration rate, CKD EPI 2021
LP-IR	Lipoprotein Insulin Resistance Index
DRI	Diabetes Risk Index

Metabolomics and lipidomics analysis were measured with nuclear magnetic resonance spectroscopy (NMR) by Labcorp® (Morrisville, USA). NMR spectra were acquired on a Vantera® Clinical Analyzer, a 400 MHz NMR instrument. The LP4 deconvolution algorithm was used to report lipoprotein particle concentrations and sizes, as well as concentrations of metabolites such as total branched-chain amino acids, valine, leucine, and isoleucine, alanine, glucose, citrate, glycine, total ketone bodies, β-hydroxybutyrate, acetoacetate, acetone. The diameters of the various lipoprotein classes and subclasses are: total triglyceride-rich lipoprotein particles (TRL-P) (24-240 nm), very large TRL-P (90-240 nm), large TRL-P (50-89 nm), medium TRL-P (37-49 nm), small TRL-P (30-36 nm), very small TRL-P (24-29 nm), total low-density lipoprotein particles (LDL-P) (19-23 nm) , large LDL-P (21.5-23 nm), medium LDL-P (20.5-21.4 nm), small LDL-P (19-20.4 nm), total high-density lipoprotein particles (HDL-P) (7.4-12.0 nm), large HDL-P (10.3-12.0 nm), medium HDL-P (8.7-9.5 nm), and small HDL-P (7.4-7.8 nm). The peak diameters for the largest (H7) to the smallest (H1) of the HDL subspecies are 12.0, 10.8, 10.3, 9.5, 8.7, 7.8, and 7.4 nm. Mean TRL, LDL, and HDL particle sizes are weighted averages derived from the sum of the diameters of each subclass multiplied by the relative mass percentage. Linear regression against serum lipids measured chemically in an apparently healthy study population (n=698) provided the conversion factors to generate NMR-derived concentrations of total cholesterol (TC), triglycerides (TG), TRL-TG, TRL-C, LDL-C, and HDL-C. NMR-derived concentrations of these parameters are highly correlated with those measured by standard chemistry methods. Lipoprotein Insulin Resistance Index (LP-IR) (0-100; least to most insulin resistant), the Diabetes Risk Index (DRI) (1-100; lowest to the highest risk of type 2 diabetes) ([2]).

Supplementary Table 3.
Total GDF-15 correlations with lipids and metabolites at baseline (Study 1 and 2; n=28) with and without FDR adjustments

Nightingale Technology				
**Molecule**	**Unadjusted**	**FDR corrected unadjusted**	**Adjusted**	**FDR corrected adjusted**
XXL-VLDL-CE	0.101	0.115	**0.027**	**0.037**
XXL-VLDL-C_%	**0.039**	0.106	**0.004**	**0.015**
XXL-VLDL-CE_%	**0.048**	0.106	**0.006**	**0.020**
XL-VLDL-C	0.137	0.146	**0.033**	**0.039**
XL-VLDL-CE	0.093	0.110	**0.017**	**0.032**
XL-VLDL-L	0.147	0.152	**0.033**	**0.039**
XL-VLDL-TG	0.133	0.146	**0.028**	**0.037**
XL-VLDL-C_%	0.079	0.106	**0.012**	**0.027**
XL-VLDL-CE_%	**0.029**	0.106	**0.003**	**0.013**
XL-VLDL-TG_%	**0.030**	0.106	**0.002**	**0.010**
L-VLDL-C	0.091	0.111	**0.019**	**0.034**
L-VLDL-CE	0.071	0.106	**0.010**	**0.027**
L-VLDL-C_%	**0.029**	0.106	**0.001**	**0.006**
L-VLDL-CE_%	0.051	0.106	**0.001**	**0.006**
L-VLDL-FE_%	0.075	0.106	**0.013**	**0.027**
L-VLDL-PL_%	**0.035**	0.106	**0.001**	**0.006**
L-VLDL-TG_%	0.079	0.106	**0.001**	**0.006**
M-VLDL-C	0.069	0.106	**0.024**	**0.035**
M-VLDL-CE	0.065	0.106	**0.022**	**0.034**
M-VLDL-FC_%	0.083	0.107	**0.022**	**0.034**
S-VLDL-C	0.055	0.106	**0.038**	**0.042**
S-VLDL-FC	**0.039**	0.106	**0.021**	**0.034**
S-VLDL-L	0.057	0.106	**0.036**	**0.041**
S-VLDL-P	0.063	0.106	**0.040**	**0.042**
S-VLDL-PL	0.079	0.106	**0.046**	**0.048**
XS-VLDL-TG	**0.041**	0.106	**0.012**	**0.027**
TG/PG	0.064	0.106	**0.032**	**0.039**
Glycerol	**0.003**	0.093	**0.000**	**0.000**
C20:0	**0.029**	0.106	**0.011**	**0.027**
Glycine	0.502	0.502	**0.008**	**0.025**
Phenylalanine	**0.015**	0.106	**0.049**	**0.049**
**Labcorp Technology**				
**Molecule**	**Unadjusted**	**FDR corrected unadjusted**	**Adjusted**	**FDR corrected adjusted**
TG	**0.022**	**0.022**	**0.028**	**0.028**
TRLC	**0.000**	**0.000**	**0.000**	**0.000**
TRLP	**0.002**	**0.004**	**0.001**	**0.002**
VS-TRLP	**0.003**	**0.004**	**0.003**	**0.004**

After logarithmic transformation, the p-value of Pearson’s correlations (Unadjusted) and partial correlations (Adjusted) by the presence of the H202D variant between total GDF-15 with Nightingale lipid and metabolite particles at baseline are presented. Significant p-values (<0.05) are highlighted in bold.

For the analysis, baseline values from participants in both Study-1 and Study-2 were used and combined in one analysis. For Study-1, since each participant underwent three separate interventions, we used the average of the three intervention baselines as a baseline value. The q-values were adjusted for false discovery rate (FDR) per the Benjamini-Hochberg Procedure (FDR corrected unadjusted and adjusted).

Supplementary Table 4.

4.a Total and H-specific GDF-15 ELISAs' precision and inter-assay coefficient variation (CV) assessed in the Mantzoros Laboratory, Boston, MA.

Total GDF15	Mean Conc (pg/mL)	Within run		Between run		Total	
		SD	CV	SD	CV	SD	CV
**Control I**	132.77	20.64	6.70%	14.43	5.40%	23.71	6.50%
**Control II**	756.59	89.38	6.30%	71.97	6.45%	86.04	6.40%

H-specific	Mean Conc (pg/mL	Within run		Between run		Total	
		SD	CV	SD	CV	SD	CV
**Control I**	133.74	34.45	9.60%	21.14	8.10%	32.96	9.25%
**Control II**	868.96	73.85	7.60%	73.1	6.70%	76.42	7.38%

Assays' precision was independently assessed in the Mantzoros Laboratory by estimating the inter-assay coefficient variation (CV) using two controls concentrations in each plate, one at the low range and one at the high range of the assay, for a total of 35 times per control in the same run. Ansh laboratories have independently performed their own extensive evaluations of the assays that can be found in the link https://www.anshlabs.com/wp-content/uploads/inserts/AL1018.pdf, accessed on January 2024.

4.b Total and H-specific GDF-15 ELISAs' external and independent validation with the Sino Biologicals (Cat:10936-H07Y) antigen epitope in the Kelesidis Laboratory, Los Angeles, CA.

Total GDF-15

Peptide concentration ng/ml	Mean OD (450 nm)	CV% (Mean, range)
300	2.007	4.15%
100	0.958	10.31%
33.3	0.319	8.65%
11.1	0.147	11.87%
3.7	0.065	9.61%
1.2	0.052	6.67%
0	0.040	-

H-specific GDF-15

Peptide concentration (ng/ml)	Mean OD (450 nm)	CV% (Mean, range)
300	2.230	13.00%
100	1.055	9.47%
33.3	0.378	13.58%
11.1	0.111	10.39%
3.7	0.048	7.38%

1.2	0.050	1.07%
0	0.040	-

The GDF-15 peptide from Sino Biologicals includes the sequence Ala 197-Ile 308. The Ansh ELISA assay is specific to Histidine at 202aa in the GDF-15 sequence and does not detect histidine 202 to aspartate mutation (DD variant). The assays' precision was performed by estimating the intra-assay coefficient variation (CV) 8 serial dilutions of a specific peptide concentration using 3 technical replicates for each dilution. For both total and H-specific GDF-15 assays, intraassay CVs were lower or equal to 13.58%, thus within an acceptable range (CV<15%). The Ansh GDF-15 ELISA reliably detected the GDF-15 peptide with acceptable linearity based on serial dilutions (1:3) within the dynamic range of the Ansh GDF-15 ELISA kit (OD 450 nm 0.05-2.230). The affinity of the Ansh GDF15 antibody and the Ansh GDF15-H specific antibody were similar for the Sino GDF15 peptide, as shown by the consistency in reproducible dose-dependent detection for total and H-specific GDF-15.

4.c Accuracy of Ansh Total GDF-15 assay assessed by Kelesidis Laboratory

Sample ID	Dilution	Expected (ng/mL)	Observed (pg/mL)	% Recovery	Average % Recovery
GDF-15 peptide	**Neat**	300	NA	NA	97.4%
	**1:3**	100	107	107%	
	**1:6**	33.3	32.6	97.9%	
	**1:9**	11.1	10.3	92.8%	
	**1:27**	3.7	3.4	91.9%	

Recovery percentage on individual samples, with multiple dilutions of GDF-15 peptide from Sino Biologicals performed in Calibrator A/sample diluent.

4.d Linearity of Ansh Total GDF-15 assay assessed by the Mantzoros Laboratory, Boston, MA.

Total GDF-15	Dilution	Expected (pg/mL)	Observed (pg/mL)	% Recovery	Average Recovery
Initial Concentration	Initial	2600	2563.04	98.58	99.36
	1:2	1300	1324.43	101.88	
	1:4	650	661.31	101.74	
	1:8	325	322.21	99.14	
	1:16	162.5	157.99	97.22	
	1:32	81.25	79.29	97.59	

H-specific GDF-15	Dilution	Expected (pg/mL)	Observed (pg/mL)	% Recovery	Average Recovery
Initial Concentration	Initial	2680	2646.85	98.76	100.37
	1:2	1340	1340.56	100.04	
	1:4	670	659.32	98.41	
	1:8	335	343.50	102.54	
	1:16	167.5	167.73	100.14	
	1:32	83.75	85.70	102.33	

Linearity of the assays with dilutions of an initial concentration of 2600 pg/mL. The % recovery found on individual samples are represented.

Supplementary Table 5.
Total and H-specific GDF-15 ELISAs evaluation on synthetic dimers of human GDF-15 homozygote for wild type (HH), heterozygote HD, and the homozygous variant (DD), assessed and publicly available by Ansh Lab.
Total GDF-15 ELISA

Total GDF-15 Assay	Expected GDF-15 Concentration (pg/mL)	Observed GDF-15 Concentration (pg/mL)	% Recovery	% Average Recovery
HH/ Wild type	500	529.9	106.0	99.5%
	50	46.5	93.1	
HD, DH, HH, DD/ Heterodimer	500	616	123.3	115.5%
	50	53..9	10.7.7	
DD/H202D Homozygous variant	500	540.0	108.0	96.4%
	50	42.4	84.8	

H-Specific GDF-15 ELISA

H-Specific GDF-15 Assay	Expected GDF-15 Concentration (pg/mL)	Observed GDF-15 Concentration (pg/mL)	% Recovery	% Average Recovery
HH/ Wild type	500	491.2	98.2	98.2%
HD, DH, HH, DD/ Heterodimer	500	154.3	30.9	30.9%
DD/H202D Homozygous variant	500	<31.1	ND	ND

More information available online: https://www.anshlabs.com/product/gdf-15-h2o2d-non-detectable-elisa/; https://www.anshlabs.com/wp-content/uploads/collateral/GDF-15_POS.pdf ([3]).

References:

1. Chrysafi, P. *et al.* Leptin alters energy intake and fat mass but not energy expenditure in lean subjects. *Nat. Commun.* **11**, 5145 (2020).
2. Matyus, S. P. *et al.* NMR measurement of LDL particle number using the Vantera Clinical Analyzer. *Clin. Biochem.* **47**, 203–210 (2014).
3. GDF-15 (H Specific) ELISA. *Ansh Labs* https://www.anshlabs.com/product/gdf-15-h2o2d-non-detectable-elisa/.

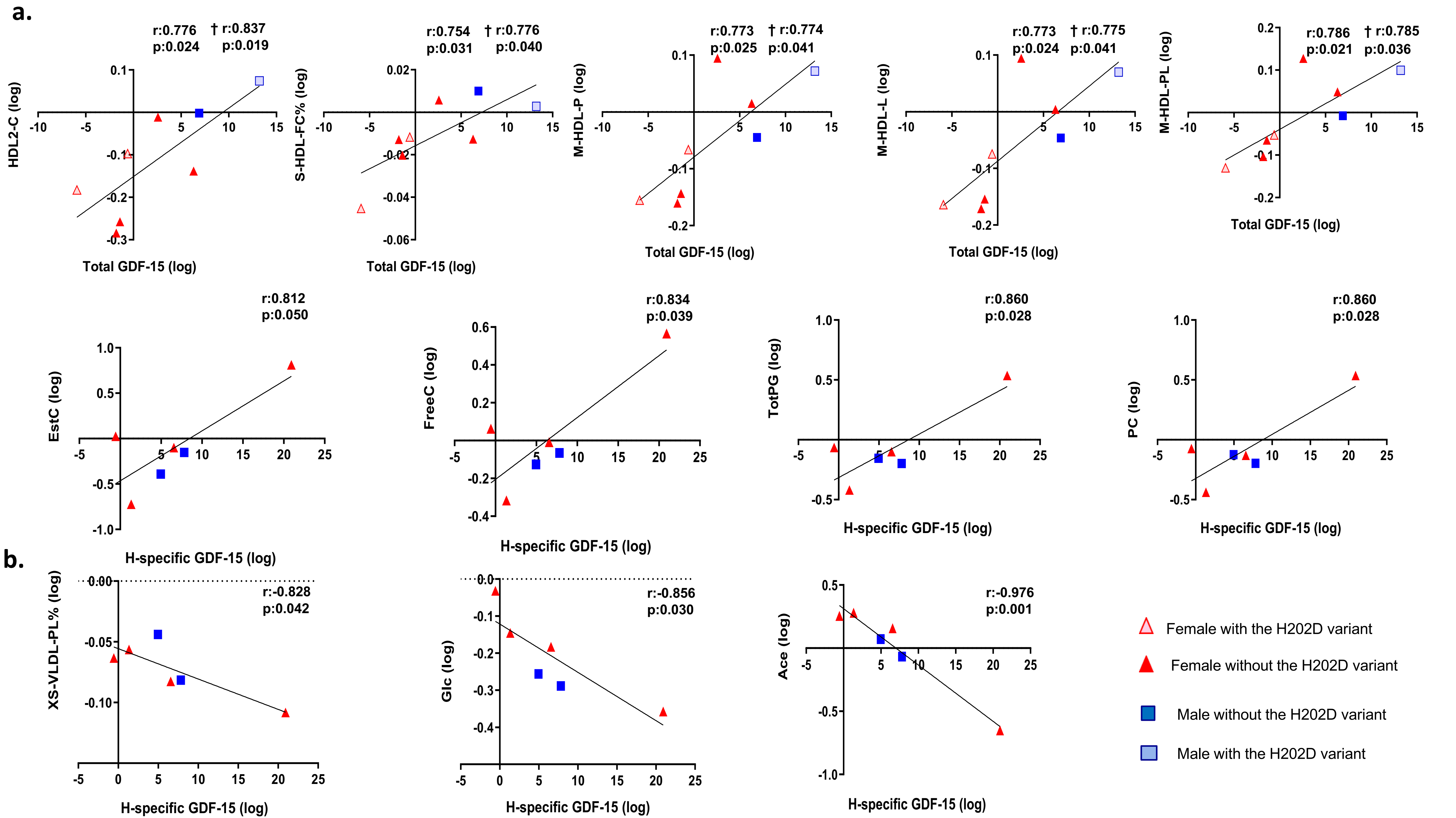
a.
r:0.776 † r:0.837
p:0.024 p:0.019
HDL2-C (log)
Total GDF-15 (log)
r:0.754 † r:0.776
p:0.031 p:0.040
S-HDL-FC% (log)
Total GDF-15 (log)
r:0.773 † r:0.774
p:0.025 p:0.041
M-HDL-P (log)
Total GDF-15 (log)
r:0.773 † r:0.775
p:0.024 p:0.041
M-HDL-L (log)
Total GDF-15 (log)
r:0.786 † r:0.785
p:0.021 p:0.036
M-HDL-PL (log)
Total GDF-15 (log)
r:0.812
p:0.050
EstC (log)
H-specific GDF-15 (log)
r:0.834
p:0.039
FreeC (log)
H-specific GDF-15 (log)
r:0.860
p:0.028
TotPG (log)
H-specific GDF-15 (log)
r:0.860
p:0.028
PC (log)
H-specific GDF-15 (log)
b.
r:-0.828
p:0.042
XS-VLDL-PL% (log)
H-specific GDF-15 (log)
r:-0.856
p:0.030
Glc (log)
H-specific GDF-15 (log)
r:-0.976
p:0.001
Ace (log)
H-specific GDF-15 (log)
Female with the H202D variant
Female without the H202D variant
Male without the H202D variant
Male with the H202D variant

Supplementary Figure 1.

Correlation of Total and H-specific GDF-15 changes with lipids and metabolites changes between days 0-3 of fasting + Placebo administration (Study 1: n=13)

Pearson's' correlation of Total and H-specific GFD-15 with Nightingale lipids and metabolites after logarithmic transformation. Points correspond to changes between days 0 and 3 (Delta) of subjects in acute complete starvation and receiving a placebo. The second set of R coefficient and two-sided p-value marked with the symbol † indicates partial correlations with adjustment for the presence of the H202D variant only in the case of total GDF-15. a.Positive correlations, b.Negative correlations. Abbreviation list: (Ace); Acetate; (EstC) Esterified cholesterol; (FreeC) Free cholesterol; (Glc) Glucose; (GDF-15) Growth differentiation factor 15; (HDL2-C) Total cholesterol in HDL2; (M-HDL-L) Total lipids in medium HDL; (M-HDL-P) Concentration of medium HDL particles; (M-HDL-PL) Phospholipids in medium HDL; (S-HDL-FC) Free cholesterol in small HDL; (PC) Phosphatidylcholine and other cholines; (TG/PG) Ratio of triglycerides to phosphoglycerides; (XS-VLDL-PL_%) Phospholipids to total lipids ratio in very small VLDL.

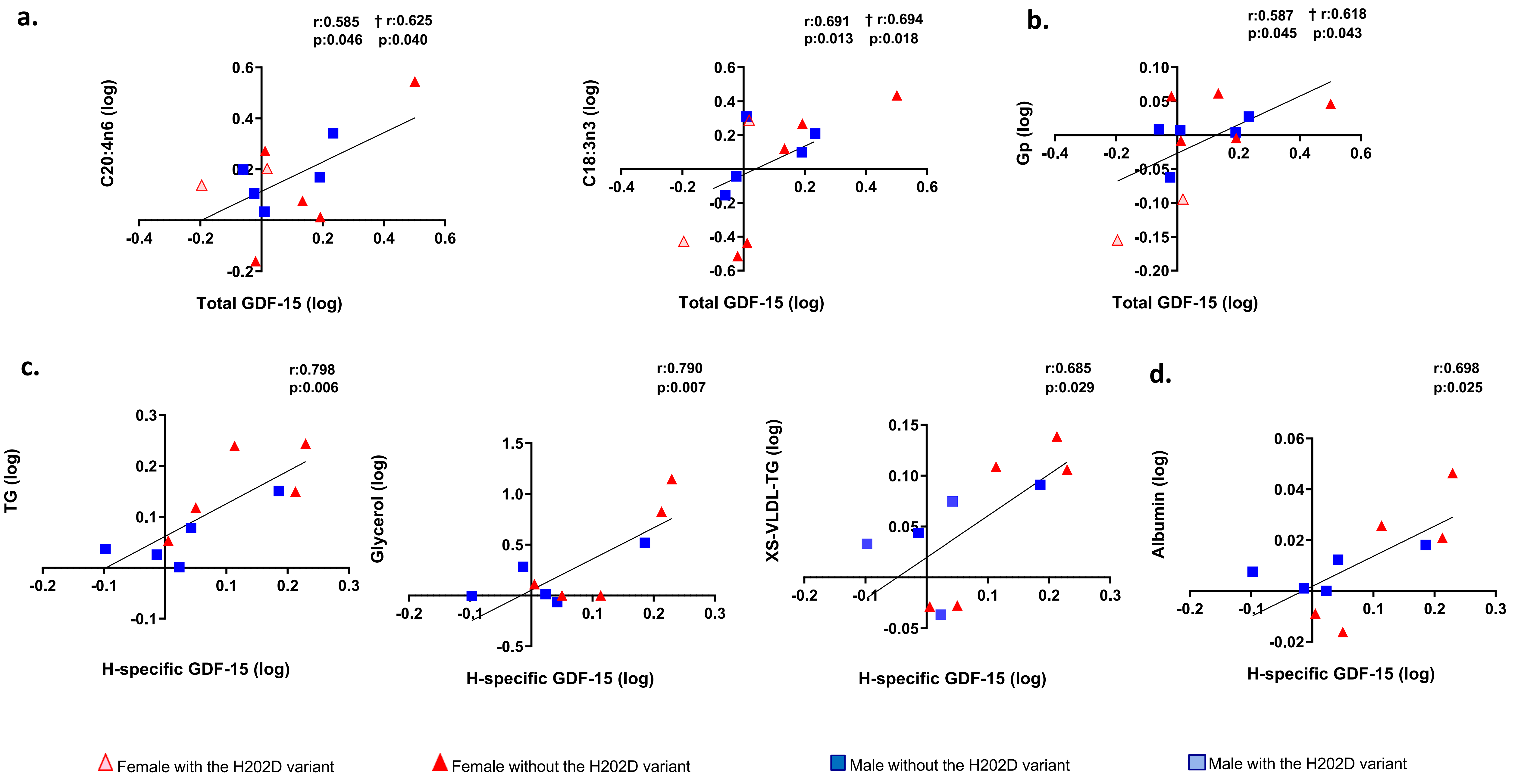

a.
r:0.585 † r:0.625
p:0.046 p:0.040
C20:4n6 (log)
Total GDF-15 (log)
r:0.691 † r:0.694
p:0.013 p:0.018
C18:3n3 (log)
Total GDF-15 (log)
b.
r:0.587 † r:0.618
p:0.045 p:0.043
Gp (log)
Total GDF-15 (log)
c.
r:0.798
p:0.006
TG (log)
H-specific GDF-15 (log)
r:0.790
p:0.007
Glycerol (log)
H-specific GDF-15 (log)
r:0.685
p:0.029
XS-VLDL-TG (log)
H-specific GDF-15 (log)
d.
r:0.698
p:0.025
Albumin (log)
H-specific GDF-15 (log)
Female with the H202D variant
Female without the H202D variant
Male without the H202D variant
Male with the H202D variant

Supplementary Figure 2.

Correlation of Total and H-specific GDF-15 changes with lipids and metabolites changes between days 0-3 of fasting + leptin replacement (Study 1: n=13)

Pearson's' correlation of Total and H-specific GFD-15 with Nightingale lipids and metabolites after logarithmic transformation. Points correspond to changes between days 0 and 3 (Delta) of subjects in acute complete starvation and receiving leptin replacement. The second set of R coefficient and two-sided p-value marked with the symbol † indicates partial correlations with adjustment for the presence of the H202D variant only in the case of total GDF-15. a.Fatty acids, b.Glycoprotein acetyls, c.Triglyceride-related particles, d.Albumin. Abbreviation list: (C20:4n6) Arachidonic acid; (C18:3n3) α-linolenic acid; (Gp) Glycoprotein acetyls; (GDF-15) Growth differentiation factor 15; (TG) Serum total triglycerides; (XS-VLDL-TG) Triglycerides in very small VLDL.

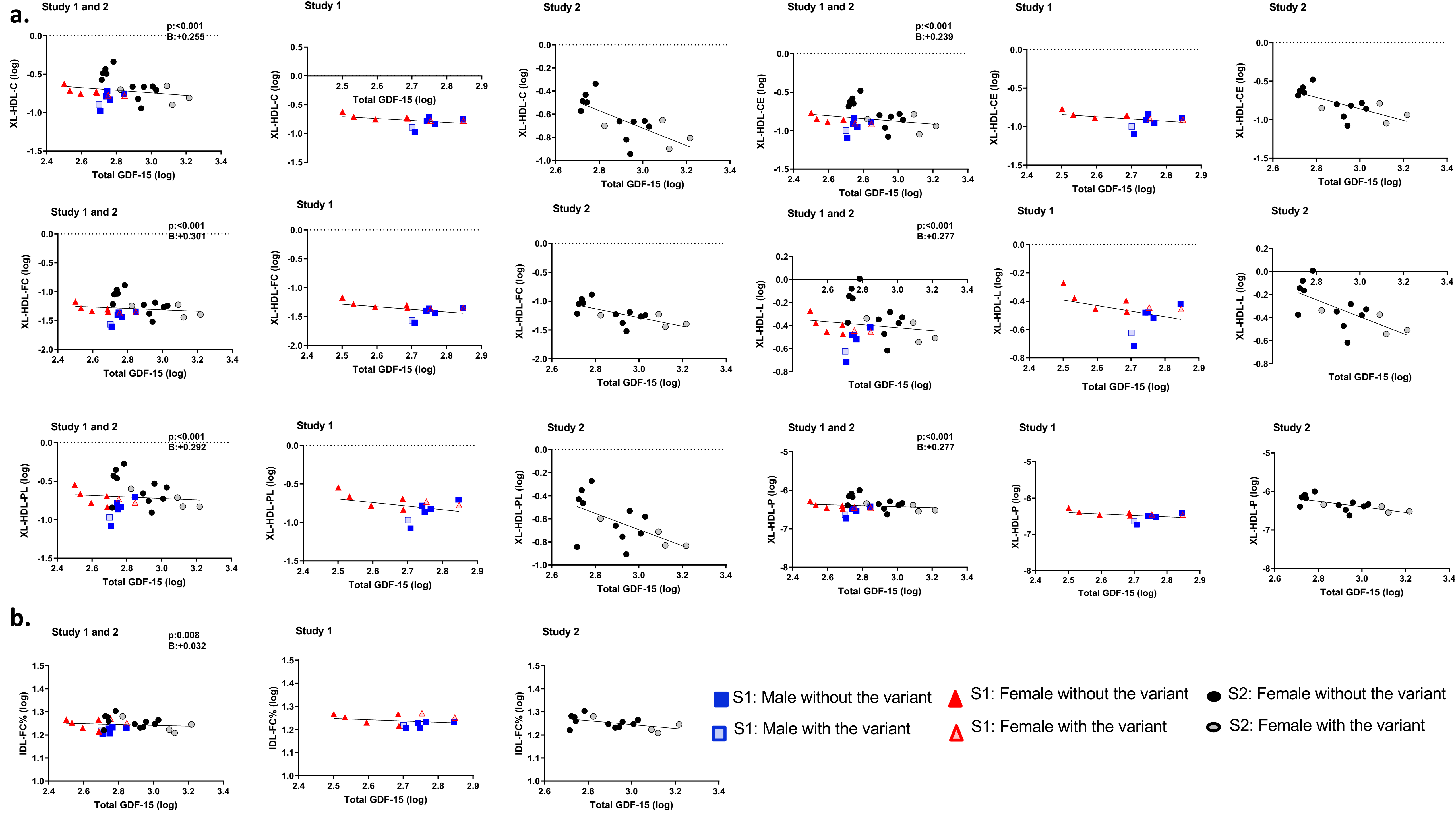
a.
Study 1 and 2
p:<0.001
B:+0.255
XL-HDL-C (log)
Total GDF-15 (log)
Study 1
XL-HDL-C (log)
Total GDF-15 (log)
Study 2
XL-HDL-C (log)
Total GDF-15 (log)
Study 1 and 2
p:<0.001
B:+0.239
XL-HDL-CE (log)
Total GDF-15 (log)
Study 1
XL-HDL-CE (log)
Total GDF-15 (log)
Study 2
XL-HDL-CE (log)
Total GDF-15 (log)
Study 1 and 2
p:<0.001
B:+0.301
XL-HDL-FC (log)
Total GDF-15 (log)
Study 1
XL-HDL-FC (log)
Total GDF-15 (log)
Study 2
XL-HDL-FC (log)
Total GDF-15 (log)
Study 1 and 2
p:<0.001
B:+0.277
XL-HDL-L (log)
Total GDF-15 (log)
Study 1
XL-HDL-L (log)
Total GDF-15 (log)
Study 2
XL-HDL-L (log)
Total GDF-15 (log)
Study 1 and 2
p:<0.001
B:+0.292
XL-HDL-PL (log)
Total GDF-15 (log)
Study 1
XL-HDL-PL (log)
Total GDF-15 (log)
Study 2
XL-HDL-PL (log)
Total GDF-15 (log)
Study 1 and 2
p:<0.001
B:+0.277
XL-HDL-P (log)
Total GDF-15 (log)
Study 1
XL-HDL-P (log)
Total GDF-15 (log)
Study 2
XL-HDL-P (log)
Total GDF-15 (log)
b.
Study 1 and 2
p:0.008
B:+0.032
IDL-FC% (log)
Total GDF-15 (log)
Study 1
IDL-FC% (log)
Total GDF-15 (log)
Study 2
IDL-FC% (log)
Total GDF-15 (log)
S1: Male without the variant
S1: Male with the variant
S1: Female without the variant
S1: Female with the variant
S2: Female without the variant
S2: Female with the variant

Supplementary figure 3.

Exploratory correlations separated per study group (Study 1 n=13, Study 2 n=15) and together (Study 1 and 2 n=28) between total GDF-15 and lipids and metabolites at baseline

Exploratory Pearson's correlations, separated per study group (Study 1, Study 2) and together (Study 1 and 2) between total GDF-15 and Nightingale lipoproteins and circulating metabolites after logarithmic transformation. R coefficient and two-sided p-value are shown, significant p-value (<0.050) indicated in bold. The variables shown are significant for effect modification. a.HDL-related particles, b.IDL-related particles. Abbreviation list: (GDF-15) Growth differentiation factor 15; (IDL-FC_%) Free cholesterol to total lipids ratio in IDL; (S1) Study 1; (S2) Study 2; (XL-HDL-C) Total cholesterol in very large HDL; (XL-HDL-CE) Cholesterol esters in very large HDL; (XL-HDL-FC) Free cholesterol in very large HDL; (XL-HDL-L) Total lipids in very large HDL; (XL-HDL-PL) Phospholipids in very large HDL; (XL-HDL-P) Concentration of very large HDL particles.

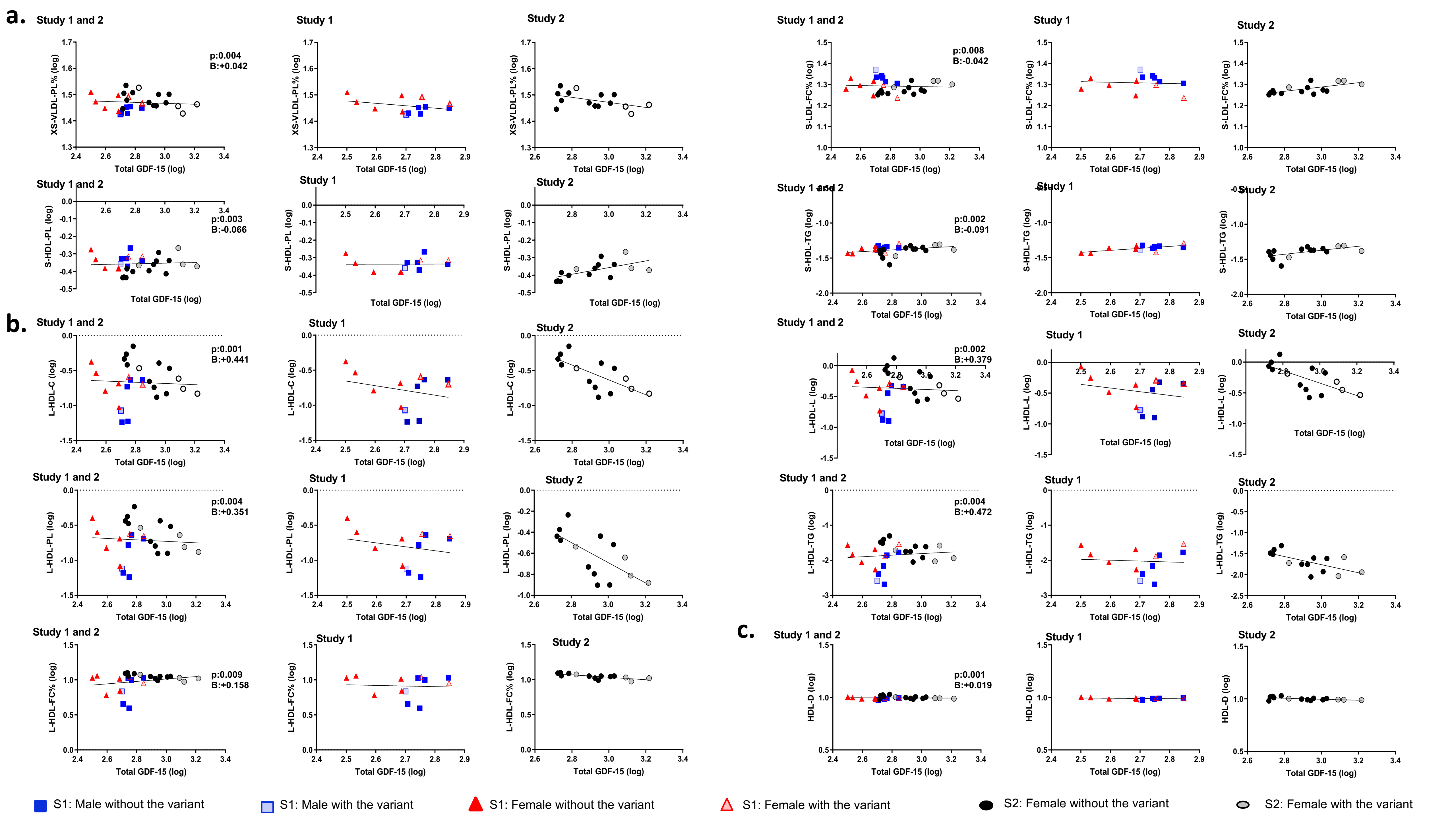
a.
Study 1 and 2
Study 1
Study 2
p:0.004
B:+0.042
XS-VLDL-PL% (log)
Total GDF-15 (log)
p:0.008
B:-0.042
S-LDL-FC% (log)
p:0.003
B:-0.066
S-HDL-PL (log)
p:0.002
B:-0.091
S-HDL-TG (log)
b.
p:0.001
B:+0.441
L-HDL-C (log)
p:0.002
B:+0.379
L-HDL-L (log)
p:0.004
B:+0.351
L-HDL-PL (log)
p:0.004
B:+0.472
L-HDL-TG (log)
p:0.009
B:+0.158
L-HDL-FC% (log)
c.
p:0.001
B:+0.019
HDL-D (log)
S1: Male without the variant
S1: Male with the variant
S1: Female without the variant
S1: Female with the variant
S2: Female without the variant
S2: Female with the variant

Supplementary figure 4.

Exploratory correlations separated per study group (Study 1 n=13, Study 2 n=15) and together (Study 1 and 2 n=28) between total GDF-15 and lipids and metabolites at baseline

Exploratory Pearson's correlations, separated per study group (Study 1, Study 2) and together (Study 1 and 2) between total GDF-15 and Nightingale lipoproteins and circulating metabolites after logarithmic transformation. R coefficient and two-sided p-value are shown, significant p-value (<0.050) indicated in bold. The variables shown are significant for effect modification. a.S and XS VLDL-related particles. b.L-HDL and HDL-related particles. Abbreviation list: (HDL-D) Mean diameter for high density lipoprotein particles; (GDF-15) Growth differentiation factor 15; (S1) Study 1; (S2) Study 2; (L-HDL-C) Total cholesterol in large HDL; (L-HDL-FC_%) Free cholesterol to total lipids ratio in large HDL; (L-HDL-L) Total lipids in large HDL; (L-HDL-PL) Phospholipids in large HDL; (L-HDL-TG) Triglycerides in large HDL; (S-HDL-TG) Triglycerides in small HDL; (S-HDL-PL) Phospholipids in small HDL; (S-LDL-FC_%) Free cholesterol to total lipids ratio in small LDL; (XS-VLDL-PL_%) Phospholipids to total lipids ratio in very small VLDL.

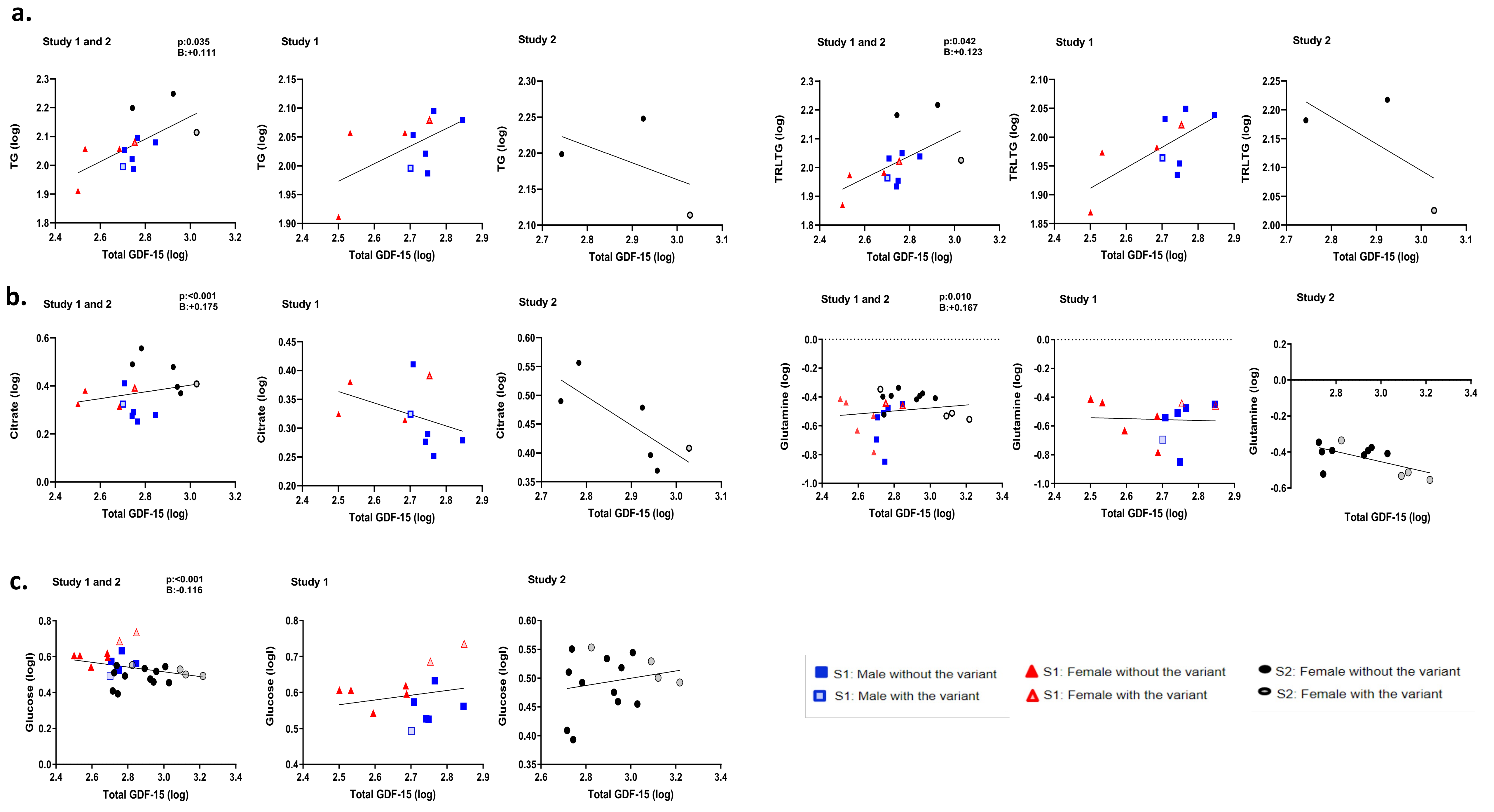
a.
Study 1 and 2
p:0.035
B:+0.111
TG (log)
Total GDF-15 (log)
Study 1
Study 2
Study 1 and 2
p:0.042
B:+0.123
TRLTG (log)
b.
Study 1 and 2
p:<0.001
B:+0.175
Citrate (log)
Study 1 and 2
p:0.010
B:+0.167
Glutamine (log)
c.
Study 1 and 2
p:<0.001
B:-0.116
Glucose (logl)
S1: Male without the variant
S1: Male with the variant
S1: Female without the variant
S1: Female with the variant
S2: Female without the variant
S2: Female with the variant

Supplementary figure 5.

Exploratory correlations separated per study group (Study 1 n=13, Study 2 n=15) and together (Study 1 and 2 n=28) between total GDF-15 and lipids and metabolites at baseline

Exploratory Pearson's correlations separated per study group and together, between GDF-15 and Nightingale and Labcorp lipoproteins and circulating metabolites included in the main Figures 4-6, which are significant for effect modification. R coefficient and two-sided p-value are shown, significant p-value (<0.050) indicated in bold. a.Triglyceride-related particles, b.Citrate and glutamine, c.Glucose. Abbreviation list: (GDF-15) Growth differentiation factor 15; (S1) Study 1; (S2) Study 2; (TG) Triglycerides; (TRLTG) TRL triglycerides.

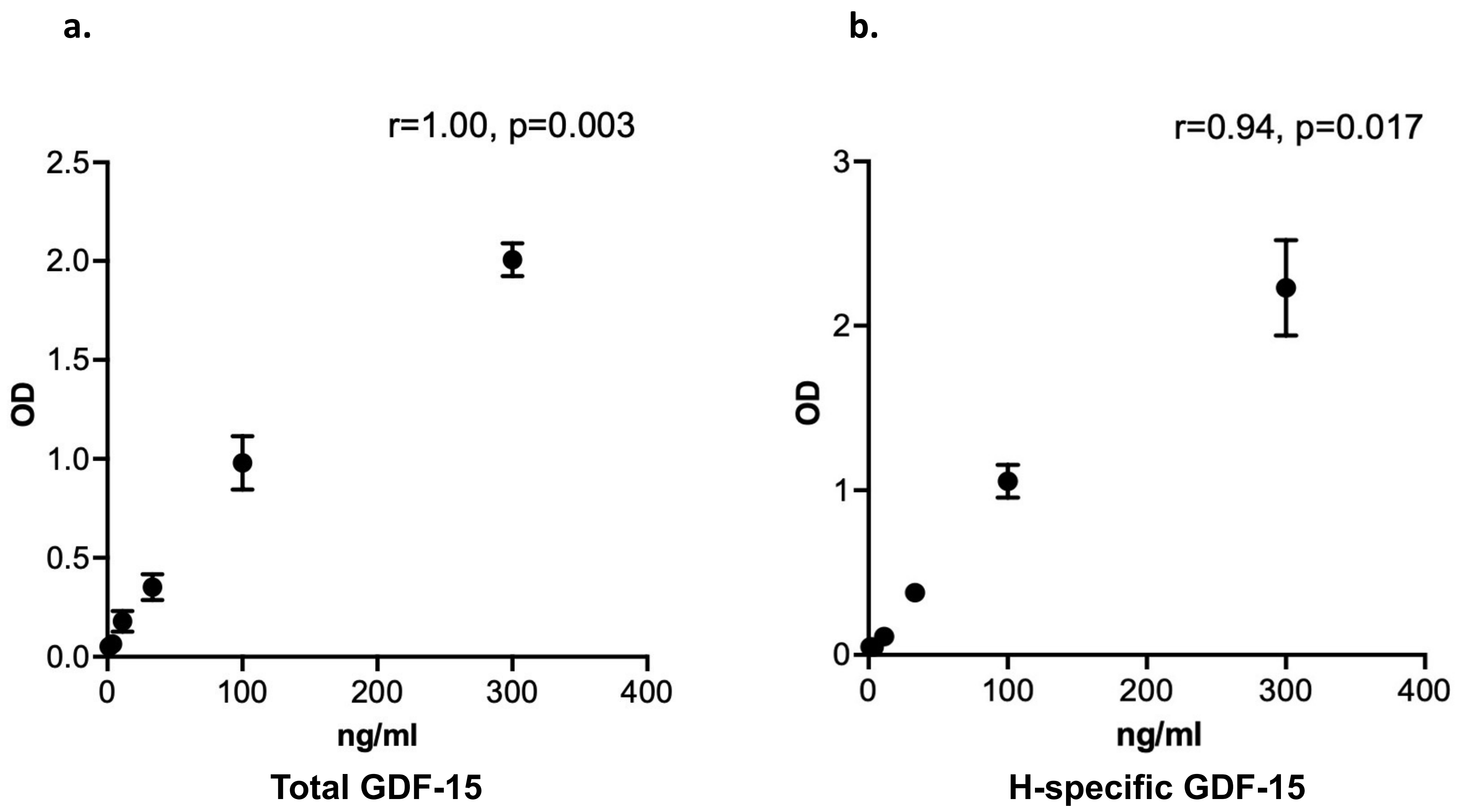
a.
r=1.00, p=0.003
OD
2.5
2.0
1.5
1.0
0.5
0.0
0
100
200
300
400
ng/ml
Total GDF-15
b.
r=0.94, p=0.017
OD
3
2
1
0
0
100
200
300
400
ng/ml
H-specific GDF-15

Supplementary Figure 6.
Validation of the commercial Ansh's ELISAs using a validated GDF-15 peptide
Total and H-specific GDF-15 peptides were measured with Ansh's Enzyme-Linked Immunosorbent Assays, as described in the methodology section. A validated GDF-15 peptide (Sino Biologicals) was serially diluted at the shown concentrations. The optical density (OD) at 450 nm (y-axis) was plotted against the estimated concentrations of the GDF-15 peptide (x-axis). The Spearman correlation coefficient and p-value are shown for both a. Total and b. H-specific GDF-15. Abbreviations: (GDF-15) Growth differentiation factor-15.
