# Supplementary material for: Total and H-specific GDF-15 levels increase in caloric deprivation independently of leptin in humans
Source: Nat Commun. 2024 Jun 18;15:5190. doi: 10.1038/s41467-024-49366-y (PMC11189399; doi:10.1038/s41467-024-49366-y)
Supplement: Supplementary file 2 — Reporting Summary [file 41467_2024_49366_MOESM2_ESM.pdf]

nature portfolio

Corresponding author(s): Christos S. Mantzoros

Last updated by author(s): May 25, 2024

Reporting Summary

Nature Portfolio wishes to improve the reproducibility of the work that we publish. This form provides structure for consistency and transparency in reporting. For further information on Nature Portfolio policies, see our Editorial Policies and the Editorial Policy Checklist.

Statistics

For all statistical analyses, confirm that the following items are present in the figure legend, table legend, main text, or Methods section.

n/a	Confirmed	
☐	☒	The exact sample size (*n*) for each experimental group/condition, given as a discrete number and unit of measurement
☐	☒	A statement on whether measurements were taken from distinct samples or whether the same sample was measured repeatedly
☐	☒	The statistical test(s) used AND whether they are one- or two-sided<br>*Only common tests should be described solely by name; describe more complex techniques in the Methods section.*
☐	☒	A description of all covariates tested
☐	☒	A description of any assumptions or corrections, such as tests of normality and adjustment for multiple comparisons
☐	☒	A full description of the statistical parameters including central tendency (e.g. means) or other basic estimates (e.g. regression coefficient) AND variation (e.g. standard deviation) or associated estimates of uncertainty (e.g. confidence intervals)
☐	☒	For null hypothesis testing, the test statistic (e.g. *F*, *t*, *r*) with confidence intervals, effect sizes, degrees of freedom and *P* value noted<br>*Give P values as exact values whenever suitable.*
☒	☐	For Bayesian analysis, information on the choice of priors and Markov chain Monte Carlo settings
☒	☐	For hierarchical and complex designs, identification of the appropriate level for tests and full reporting of outcomes
☐	☒	Estimates of effect sizes (e.g. Cohen's *d*, Pearson's *r*), indicating how they were calculated

Our web collection on statistics for biologists contains articles on many of the points above.

Software and code

Policy information about availability of computer code

Data collection	N/A
Data analysis	Statistical analysis was performed with SPSS v 28.0.1.0 (SPSS, Inc, Chicago, IL) for Windows, with GraphPad Prism 9.3.1 (GraphPad Software Inc, La Jolla, CA), R Studio (GGally package), and with MetaboAnalyst R.

For manuscripts utilizing custom algorithms or software that are central to the research but not yet described in published literature, software must be made available to editors and reviewers. We strongly encourage code deposition in a community repository (e.g. GitHub). See the Nature Portfolio guidelines for submitting code & software for further information.

Data

Policy information about availability of data

All manuscripts must include a data availability statement. This statement should provide the following information, where applicable:

- Accession codes, unique identifiers, or web links for publicly available datasets
- A description of any restrictions on data availability
- For clinical datasets or third party data, please ensure that the statement adheres to our policy

The data supporting the findings of this study are not publicly accessible owing to institutional regulations but will be made available from the corresponding author upon reasonable request and under a data sharing agreement with our institution. Relevant information on the ELISA assay used is available in Supplementary Table-4 and 5 and Supplementary Figure-6. A list of all identified metabolites is available in Supplementary Table-2.

Research involving human participants, their data, or biological material

Policy information about studies with human participants or human data. See also policy information about sex, gender (identity/presentation), and sexual orientation and race, ethnicity and racism.

Reporting on sex and gender	Sex was considered in the study's design. Study 1 included both sexes. Study 2 was only for female subjects with documented Hypothalamic amenorrhea.
Reporting on race, ethnicity, or other socially relevant groupings	For the initial trials, individuals were recruited from the Boston area using advertisement and postings through the Boston area Universities Students Offices. Subjects were recruited in proportion to their ethnic balance in the local community. Subjects were chosen without regard for racial, social, economic or other status.
Population characteristics	Study 1: Healthy lean patients. The total sample mean age was 23.61 ± 1.01 years, with mean weight 65.60 ± 3.33 kg, mean fat mass 15.10 ± 1.16 kg, and mean lean mass 50.50 ± 3.66. (men: mean: age 23.50 ± 1.54 years; weight 76.06 ± 1.72 kg, women:mean:age23.71± 1.46years; weight 56.65 ± 2.33 kg). Subjects did not have history of any illness, or medication use, that may affect the concentrations of the hormones to be studied.<br><br>Study 2: Lean females with chronic mild caloric deficit and hypothalamic amenorrhea of at least 6 months duration with low or normal LH and FSH, e.g. due to strenuous exercise (running >20 miles per week or equivalent) or low weight. The mean age was 26.13+/-1.07 years; weight 56.20 ± 1.71 kg; fat mass 13.20 ± 0.82 kg, lean mass 42.99 ± 1.33 kg. Baseline leptin <5 ng/mL. Body weight within +/- 15% of ideal body weight and stable for 6 months (no change > 5 lbs). None of the participants had a significant medical history that may affect the concentrations of the hormones to studied or ability to participate in the study.
Recruitment	For initial trials, participants were recruited from the Boston area using advertisement and postings through the Boston area Universities Students Offices., in proportion to their ethnic balance in the local community. Study protocols are uploaded in figsgare.com: http://figshare.com/s/696fe9847bc6898de577. [ClinicalTrials.gov Study-1: NCT00140231].[ClinicalTrials.gov Study-2: NCT00130117].
Ethics oversight	The humanstudies were approved by the Institutional Review Board of Beth Israel Deaconess Medical Center and were performed at the General Clinical Research Center (GCRC) in accordance with the declaration of Helsinki under an investigator-held IND. All participants obtained written informed consent before inclusion in

Note that full information on the approval of the study protocol must also be provided in the manuscript.

Field-specific reporting

Please select the one below that is the best fit for your research. If you are not sure, read the appropriate sections before making your selection.

☒ Life sciences ☐ Behavioural & social sciences ☐ Ecological, evolutionary & environmental sciences

For a reference copy of the document with all sections, see nature.com/documents/nr-reporting-summary-flat.pdf

Life sciences study design

All studies must disclose on these points even when the disclosure is negative.

Sample size	Study 1: Sample size was determined to Six healthy lean men (mean age 23.50 ± 1.01 years; mean weight 76.06 ± 1.72 kg) and seven healthy lean women (mean age 23.71 ±1.46 years; mean weight 56.65 ± 2.33 kg). Sample size was based on previously published work and on power analysis alpha=5% and 1-B=80% to detect a 50% difference in mean leptin concentration.<br><br>Study 2: The total number of evaluable subjects needed for the study was 34 (17 per treatment arm). Plan was to screen and consent up to 100 subjects in order to have 50 subjects entering the study and 34 subjects completing the study as described in the uploaded protocol. Sample size was based on previously published work and on power analysis alpha=5% and 1-B=80% such that a minimum of 4% difference in bone mineral density could be detected between groups and assuming a variance model with SD=4. In this study n=15 based on sample availability (mean age=26.13+/-1.07 years; mean weight=56.20 ± 1.71 kg).
Data exclusions	Study 1: Exclusion Criteria included a history of any illness that may affect the concentrations of the hormones to be studied, medications known toaffect the hormones to be measured, history anaphylactoid-like reactions, or a known hypersensitivity to E. coli derived proteins. Two males withdrew before completing all three phases, and one female before completing one of the phases (fasting + placebo). We excluded the two males from the analysis as their corresponding data was insufficient, but we included the female since she had completed 2/3 phases/visits, so that in total findings from 13 subjects (6 males and 7 females) were analyzed.<br><br>Study 2: Exclusion criteria included patients with significant medical history or other endocrine causes of amenorrhea or pregnancy; patients with alcoholism, drug abuse, or smoking history, an active eating disorder, depression or other psychiatric disease; subjects on medications known toaffect the hormones to be measured. None of the subjects had hyperprolactinemia, hypo- or hyperthyroidism, Cushing's syndrome, congenital adrenal hyperplasia or primary ovarian failure. One participant in the metreleptin treated group withdrew from the study because she developed injection-site reactions soon after the baseline visit.
Replication	All samples were run in duplicates within the same run for a given subject and were repeated if coefficient of variation for any sample was N15%. All attempts of replication were successful.

Randomization	Study 1: Subjects were assigned in a random order to each of the three admissions (i.e. fed, fasting+placebo, fasting+leptin) Study 2: Subjects were randomly assigned in a 1:1 fashion to receive metreleptin or placebo.
Blinding	Study 1: Investigators and participants were blinded during group allocation and data collection (double blinded) Study 2: Investigators and participants were blinded during group allocation and data collection (double blinded)

Reporting for specific materials, systems and methods

We require information from authors about some types of materials, experimental systems and methods used in many studies. Here, indicate whether each material, system or method listed is relevant to your study. If you are not sure if a list item applies to your research, read the appropriate section before selecting a response.

Materials & experimental systems

n/a	Involved in the study
☒	☐ Antibodies
☒	☐ Eukaryotic cell lines
☒	☐ Palaeontology and archaeology
☒	☐ Animals and other organisms
☐	☒ Clinical data
☒	☐ Dual use research of concern
☒	☐ Plants

Methods

n/a	Involved in the study
☒	☐ ChIP-seq
☒	☐ Flow cytometry
☒	☐ MRI-based neuroimaging

Clinical data

Policy information about clinical studies

All manuscripts should comply with the ICMJEguidelines for publication of clinical research and a completedCONSORT checklist must be included with all submissions.

Clinical trial registration	[ClinicalTrials.gov Study-1: NCT00140231].[ClinicalTrials.gov Study-2: NCT00130117].
Study protocol	http://figshare.com/s/696fe9847bc6898de577
Data collection	Study 1: Study start 2001-10; final data collection date for primary outcome measure 2011-03; study completion: 2016-12. Subjects were admitted to the General Clinical Research Center (GCRC) for a 4 day admission. Blood draw was performed in the evening of day 0 and inthe mornings of days 1,2,3, prior to any intervention. Body composition (bioelectric impedance analysis; RJL Systems, Clinton Township, MI), RMR (DeltaTrac II Metabolic Monitor; SensorMedics), and morning vital signs were assessed at the beginning and end of each study.<br><br>Study 2: Study start 2010-08; actual primary completion date 2011-08; study completion 2016-12. Subjects were admitted to the General Clinical Research Center (GCRC) for all visits. Fasting blood samples were collected every four weeks, while body composition and RMRweremeasured every12 weeks with DEXA andSensormedics Vmax Encore quipment (VIASYS Respiratory Care Inc,), respectively.
Outcomes	Total and H-specific GDF-15 increased in response to complete acute starvation in healthy lean subjects, independently of starvationinduced leptin changes. Total GDF-15 was higher in females with chronic mild caloric deficit due to relative energy deficiency in sports (REDs) than in healthy lean subjects. These changes were also independent of leptin administration. As secondary aims, baseline GDF-15 was mainly positively correlated with triglyceride-rich particles and lipoproteins, and during acute metabolic stress, GDF-15 associations with metabolites/lipids might differ in subjects with the H202D variant. GDF-15 increases with energy deprivation in humans in a leptin-independent manner, which is inconsistent with its proposed role in decreasing human body weight.

Plants

Seed stocks	*Report on the source of all seed stocks or other plant material used. If applicable, state the seed stock centre and catalogue number. If plant specimens were collected from the field, describe the collection location, date and sampling procedures.*
Novel plant genotypes	*Describe the methods by which all novel plant genotypes were produced. This includes those generated by transgenic approaches, gene editing, chemical/radiation-based mutagenesis and hybridization. For transgenic lines, describe the transformation method, the number of independent lines analyzed and the generation upon which experiments were performed. For gene-edited lines, describe the editor used, the endogenous sequence targeted for editing, the targeting guide RNA sequence (if applicable) and how the editor was applied.*
Authentication	*Describe any authentication procedures for each seed stock used or novel genotype generated. Describe any experiments used to assess the effect of a mutation and, where applicable, how potential secondary effects (e.g. second site T-DNA insertions, mosiacism, off-target gene editing) were examined.*
